# Improving success of non-communicable diseases mobile phone surveys: Results of two randomized trials testing interviewer gender and message valence in Bangladesh and Uganda

**DOI:** 10.1371/journal.pone.0285155

**Published:** 2023-05-24

**Authors:** Alain Labrique, Madhuram Nagarajan, Gulam Muhammed Al Kibria, Andres Vecino-Ortiz, George W. Pariyo, Joseph Ali, Michelle R. Kaufman, Dustin Gibson

**Affiliations:** 1 Johns Hopkins Bloomberg School of Public Health, Baltimore, MD, United States of America; 2 Makerere University College of Health Sciences, Kampala, Uganda; University of California Davis School of Medicine, UNITED STATES

## Abstract

**Introduction:**

Although interactive voice response (IVR) is a promising mobile phone survey (MPS) method for public health data collection in low- and middle-income countries (LMICs), participation rates for this method remain lower than traditional methods. This study tested whether using different introductory messages increases the participation rates of IVR surveys in two LMICs, Bangladesh and Uganda.

**Methods:**

We conducted two randomized, controlled micro-trials using fully-automated random digit dialing to test the impact of (1) the gender of the speaker recording the survey (i.e., survey voice); and (2) the valence of the invitation to participate in the survey (i.e., survey introduction) on response and cooperation rates. Participants indicated their consent by using the keypad of cellphones. Four study arms were compared: (1) male and informational (MI); (2) female and information (FI); (3) male and motivational (MM); and (4) female and motivational (FM).

**Results:**

Bangladesh and Uganda had 1705 and 1732 complete surveys, respectively. In both countries, a majority of the respondents were males, young adults (i.e., 18-29-year-olds), urban residents, and had O-level/above education level.

In Bangladesh, the contact rate was higher in FI (48.9%), MM (50.0%), and FM (55.2%) groups than in MI (43.0%); the response rate was higher in FI (32.3%) and FM (33.1%) but not in MM (27.2%) and MI (27.1%). Some differences in cooperation and refusal rates were also observed. In Uganda, MM (65.4%) and FM (67.9%) had higher contact rates than MI (60.8%). The response rate was only higher in MI (52.5%) compared to MI (45.9%). Refusal and cooperation rates were similar.

In Bangladesh, after pooling by introductions, female arms had higher contact (52.1% vs 46.5%), response (32.7% vs 27.1%), and cooperation (47.8% vs 40.4%) rates than male arms. Pooling by gender showed higher contact (52.3% vs 45.6%) and refusal (22.5% vs 16.3%) rates but lower cooperation rate (40.0% vs 48.2%) in motivational arms than informational arms. In Uganda, pooling intros did not show any difference in survey rates by gender; however, pooling by intros showed higher contact (66.5% vs 61.5%) and response (50.0% vs 45.2%) rates in motivational arms than informational arms.

**Conclusion:**

Overall, we found higher survey rates among female voice and motivational introduction arms compared to male voice and informational introduction arm in Bangladesh. However, Uganda had higher rates for motivational intro arms only compared to informational arms. Gender and valence must be considered for successful IVR surveys.

**Trial registration:**

Name of the registry: ClinicalTrials.gov. Trial registration number: NCT03772431. Date of registration: 12/11/2018, Retrospectively Registered. URL of trial registry record: https://clinicaltrials.gov/ct2/show/NCT03772431?term=03772431&cond=Non-Communicable+Disease&draw=2&rank=1. Protocol Availability: https://www.researchprotocols.org/2017/5/e81.

## Introduction

Over the past several decades, survey research has been challenged by the exponential increase in the volume of digital traffic vying for attention [1]. Whether internet, email, or phone-based–surveys struggle to capture and hold a putative respondent for even a few minutes, fighting against a supersaturation of digital requests and so-called “spam” commercial messages and calls [2,3].

The use of high-volume commercial robocalling around the world has led to a distrust of and non-response to random calls from unknown numbers and proliferation of spam blockers [2,3]. Many commercial and clinical interactions are followed by a set of questions or require a post-experience quality assessment, rapidly giving rise to “survey exhaustion” [4]. From the intentional masking of phone numbers to seem authentic to the use of catchy images or messages, a plethora of strategies, both genuine and nefarious, have been attempted in efforts to increase engagement and participation [5,6].

Despite these obstacles, interactive voice response (IVR) surveys remain popular and important tools for public health, relying on automated calls to ask respondents to press keypad numbers in answer to questions about their risk factors, health, and environment [7,8]. National public health surveys such as the Behavioral Risk Factor Surveillance System (BRFSS) in the United States compete with the high number of daily spam calls, thus struggling to meet their sample sizes [9,10]. Data from the Pew Research Center, a research group that conducts the General Social Survey in the U.S., shows a steady decline in phone survey response rates since the late 1990s, with response rates previously as high as 36% dropping to less than 10% in 2016 [11].

While many low- and middle-income countries (LMICs) have “leapfrogged” the fixed phone line and directly entered the cellular era [12], comparable challenges of low survey response rates confront researchers conducting mobile phone surveys in LMIC settings. Despite this jump directly to mobile phones, the challenges of unwanted calls and messages remain [13]. A 2019 study of the global burden of these “spam” messages to mobile phones found an average of 119 unwanted text messages per month going to users in Ethiopia, to 27 and 61 per month going to users in Bangladesh and India, respectively [14].

Drawing on intrinsic and extrinsic motivators to increase responses to surveys is a well-researched area [15]. One approach is to position research participation as an important societal responsibility, another is to motivate survey participation through financial incentives or rewards [16–18]. Cash incentives have been shown to be successful in reducing non-response in mobile phone surveys (MPS) as well as attrition rates in medication adherence [17,19–22]. A meta-analysis quantifying the dose-response relationship between incentives and response rates in household surveys (whether via mail, phone, or in-person) found a nonlinear relationship between incentives and an increased response rate [23]. Mercer et al. reported that “…respondents value nonmonetary incentives differently than monetary incentives. Gifts may have other characteristics beyond their perceived cash value that determine their worth” [22].

In the broader literature on postal and landline-based telephone surveys, innovations range from the use of emotional motivation to the selection of culturally appropriate voices [21,23]. Some of the evidence for the effectiveness of content that draws on emotional drivers to capture and retain respondents is borrowed from advertising research and behavioral interventions [24]. Programs such as *Swacch Bharat* (translated as “Clean India”) feature nostalgia-inducing advertising by celebrities to promote civic engagement; this approach leverages patriotic/group pressure to trigger action. This has not, however, been well-tested in phone-based surveys [25].

The perceived gender of the recorded voice may also play a role in capturing initial respondents as well as in their willingness to answer sensitive questions. Further, the relative effectiveness of male versus female voices may vary by the gender of the respondent, context, and subject matter [26]. This effectiveness (or lack thereof) could have implications for the completeness of data and minimizing gender data gaps in mobile data collection efforts. Though differential effects of the interviewer’s voice and perceived gender, across respondent gender, have been reported in other studies [27,28], few studies investigated the combined impact of gender and type of introductory message on mobile phone survey performance, especially in LMICs. The ubiquitous growth of mobile phones in LMICs has been accompanied by the potential to use this platform for public health data collection, however, understanding the impact of introductory messages will be helpful to design and implement MPS in a shorter time and at a lower cost. The study aims to fill these gaps in knowledge and assess how different introductory message characteristics (i.e., gender of speaker recorded and valence of the introductory message) affect survey performance metrics of a non-communicable disease (NCD)-risk factor IVR questionnaire in Bangladesh and Uganda.

## Methods

### Study design

**Fig 1:**
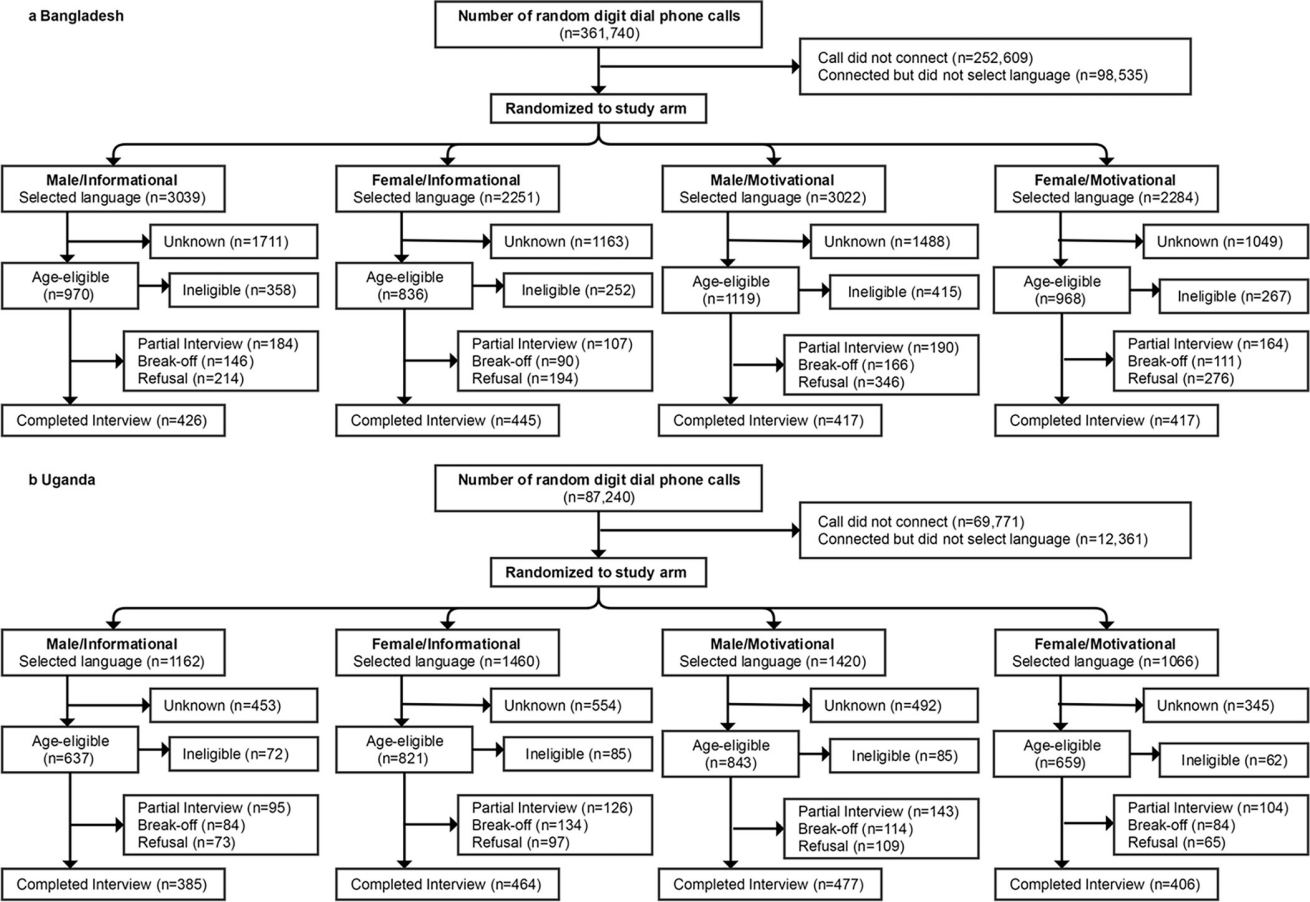
Consolidated Standard of Report Trial Diagram of the Study Arms Enrollment in Bangladesh (1a) and Uganda (1b).

We conducted two randomized controlled micro-trials to test ways to improve the performance of IVR MPS in Bangladesh and Uganda. As indicated in Fig 1, participants were randomized to one of four arms with messages varying by male or female voice, and whether the invitation to participate in the study was paired with information or motivational messaging. Four arms were included in both countries: 1) male voice, informational introduction (MI); 2) female voice, informational introduction (FI); 3) male voice, motivational introduction (MM); and 4) female voice, motivational introduction (FM). We also used pooling, which generated two arms across introductory messages: 1) male voice (MV) and 2) female voice (FV); and survey voices: 1) informational intro (II) and 2) motivational intro (MI). The allocation ratio was 1:1:1:1.

### Randomization and masking

Study participants were sampled using the random digit dialing (RDD) method. Participants who received the call and connected to the IVR system were screened for age greater or equal to 18 years old to determine participation eligibility. Age-eligible participants were screened for willingness to participate in the study (answer survey questions), indicated by response choice on their mobile phones. Eligibility- and consent-screened participants were presented with the remainder of the IVR survey [29].

A fully automated pseudo-random number generator was used to produce random numbers of seven-digit length, prefixed with (randomly generated) mobile network operator-specific three-digit phone codes and the country codes for Bangladesh (880) and Uganda (256). Participants were randomized to their respective study arms after choosing their preferred survey language, and the randomization was through an automated process built into the IVR platform by the provider. Study participants could not be masked to their study group allocation due to the nature of the intervention, namely different genders of voices and different messages on the recordings played to the participants [30]. The data cleaning was done by researchers blinded to the allocation of study participants.

### Procedure

The study procedures for Bangladesh and Uganda were comparable. Participants who picked up the phone call were asked to indicate language preference through a numeric response on the keypad. In Bangladesh, the languages available were Bangla and English; in Uganda, the languages available were Luganda, Luo, Runyakitara, and English. In both countries, the languages offered covered the majority of the population.

Survey questions were administered in the following order of broad categories: 1) language selection; 2) introduction to the survey, including the description of the requirements to be met in order to receive an incentive; 3) screening questions (age-eligibility, consent); 4) demographic questions; and 5) five NCD modules. The NCD modules are related questions grouped as they pertain to 1) tobacco use, 2) alcohol use, 3) diet, 4) physical activity, and 5) high blood pressure and diabetes markers. While the order of administration of different NCD modules was randomized to minimize bias due to drop-off or attrition mid-survey, skip patterns within modules were preserved by keeping the order of questions in each NCD module constant. After confirming the age, eligible participants were asked to provide consent by pressing number 1 button on mobile phone’s screen.

The questions in each module in the IVR survey were based on standardized questions from surveys such as the World Health Organization’s STEPwise surveillance [31] and Tobacco Questions for Surveys (TQS) [32]. They were chosen by experts in NCDs and compiled in an initial questionnaire. Cognitive understanding and usability were initially tested at Johns Hopkins University, with persons who identified as being from a low- or middle-income country setting [33]. A series of key informant interviews (KIIs), focus group discussions (FGDs), and user groups were conducted in each country, and feedback was used to refine and adapt the questionnaire and deployment to the country context [34]. Audio files of the questionnaire were tested at the country level prior to survey deployment to ensure that translations and recordings were comprehensible.

The IVR surveys were deployed between 08:00 AM and 08:00 PM local time with a single attempt made to contact each randomly generated number. Survey participants could choose to repeat questions through key presses on their mobile phones as they moved through the survey. Participants did not incur charges for the airtime taken to complete the survey and were informed of this; those who completed the IVR survey would receive a small airtime incentive delivered through their provider. The scripts of the motivational and informational intros are shown in S1 Table.

The two micro-trials generated 361,740 calls with 1,705 surveys completed in Bangladesh (Fig 1A), and 87,240 calls placed with 1,732 complete surveys in Uganda (Fig 1B). These trials ran between March and April of 2017 in Uganda and June and July of 2017 in Bangladesh. The number of participants in MI, FI, MM, and FF was, respectively, 426, 445, 417, and 417 in Bangladesh, and 385, 464, 477, and 403 in Uganda.

### Outcomes

Survey outcomes (i.e., survey performance metrices) were categorized using standard definitions of disposition codes and survey rates from the American Association for Public Opinion Research (AAPOR) [35]. The definitions of the disposition codes as used in this study and equations for primary and secondary outcomes are listed in Table 1.

**Table 1:** AAPOR definitions and equations.

Disposition Codes		Definition/Equations
**AAPOR Definitions**	Complete Interviews (I)	Participants who answered at least 5 of the 7 modules.
	Partial Interviews (P)	Participants who answered 2,3, or 4 modules.
	Refusals (R)	Participants who either did not press a button on their mobile phone to indicate consent, refused consent, or who hung-up at the consent question.
	Break-offs (R)	Participants who consented but did not complete a module other than demographics
	Unknown (U)	Participants who selected a survey language but did not answer the age question, includes unknown household (UH) and unknown other (UO).
	Estimated Unknown (eU)	Estimated proportion of unknown cases that were age eligible.
	Ineligible on age	Participant who indicated an age less than 18 years of age.
	Other (O)	Other than any of the above
**AAPOR Equations**	Contact Rate #2	$\frac{\left(I+P+R+O\right)}{I+P+R+O+e(UH+UO)}$
	Response Rate #4	$\frac{\left(I+P\right)}{I+P+R+O+e(UH+UO)}$
	Refusal Rate #2	$\frac{\left(R\right)}{I+P+R+O+e(UH+UO)}$
	Cooperation Rate #1	$\frac{\left(I\right)}{I+P+R+O}$

Abbreviation: AAPOR: American Association for Public Opinion Research.

The primary outcomes of the study were the response rate and the cooperation rate. The response rate was the sum of *complete and partial interviews* divided by the sum of all possible eligible participants (i.e., complete and partial interviews, refusals, breakoffs, and the estimated proportion of age-eligible unknowns). The cooperation rate was the number of *complete interviews* divided by the sum of complete and partial interviews, refusals, and others. The secondary outcomes were the contact rate and refusal rate. The contact rate is the sum of complete and partial interviews, refusals, and others divided by the sum of all possible eligible participants; the refusal rate is the number of refusals over that same denominator (Table 1).

### Statistical analysis

Sample sizes for Bangladesh and Uganda were calculated under the assumptions of a control arm cooperation rate of 30%, an alpha of 0.05, and a power of 80%. This calculation meant 376 completed survey responses were needed in each arm to detect a 10% absolute difference in cooperation rates between two study arms. We did not inflate sample sizes for multiple comparisons, as per the recommendation by Rothman [36].

Demographic characteristics of the participants with complete interviews and disposition codes of all phone calls were compared across the four study arms in both countries. Log-binomial regression was used to calculate the risk ratio (RR) and corresponding 95% confidence interval (CI) for contact, response, refusal, and cooperation rates, using the male voice and informational introduction as the reference group for the four study arms. Data were pooled by survey voice and survey introduction to compare the isolated effects of survey voice and survey introduction on contact, response, refusal, and cooperation rates (RR and 95% CI) in both countries. Sensitivity analyses were run comparing respondent demographics of complete and partial interviews, and using different definitions of the outcome rates, calculated without applying *e* for the unknown participants (Table 1). Analyses were conducted using Stata/SE (version 14.1; Stata Corp, College Station, TX, USA) [37]. An alpha of 0.05 was assumed for all tests of statistical significance.

### Ethical approval and consent to participate

Ethical clearance was received from the institutional review boards of the institutions involved in the micro-trials, namely the Johns Hopkins University Bloomberg School of Public Health, Baltimore, Maryland, U.S.; Makerere University School of Public Health, Uganda; the Uganda National Council for Science and Technology, Uganda; and the Institute of Epidemiology Disease Control and Research, Bangladesh. Eligible study participants had to provide informed consent to participate using keypad of cell phones. After confirming the age by pressing mobile phone numbers, eligible participants were provided the consent disclosure statement. They were asked to provide consent by pressing the 1 button on the mobile phone.

## Results

In Bangladesh, the demographic characteristics of complete interviews were balanced across the 4 arms (Table 2). A majority of the study participants were male (84.7%-87.8%) between the ages of 18–29 years (64.6% - 68.1%). The most common levels of education reported were between O-level (24.5% - 29.9%), and A-level (22.3% - 25.8%) equivalents. About half the respondents were from an urban location (47.7% - 56.6%), and almost all took the survey in Bangla (over 98%).

**Table 2:** Demographics of complete interviews by study arm in Bangladesh and Uganda, n (%).

Demographics	Bangladesh				Uganda			
	Informational Intro		Motivational Intro		Informational Intro		Motivational Intro	
	Male Voice (n = 426)	Female Voice (n = 445)	Male Voice (n = 417)	Female Voice (n = 417)	Male Voice (n = 385)	Female Voice (n = 464)	Male Voice (n = 477)	Female Voice (n = 406)
Gender								
Male	361 (84·7)	389 (87·4)	360 (86·3)	366 (87·8)	293 (76·1)	358 (77·2)	354 (74·2)	314 (77·3)
Female	59 (13·9)	55 (12·4)	53 (12·7)	48 (11·5)	92 (23·9)	106 (22·8)	123 (25·8)	92 (22·7)
Transgender	6 (1·4)	1 (0·2)	4 (1·0)	3 (0·7)	-	-	-	-
Age (year)								
18–29	280 (65·7)	301 (64·6)	281 (67·4)	284 (68·1)	278 (72·2)	319 (68·8)	347 (72·8)	285 (70·2)
30–49	115 (27·0)	123 (27·6)	104 (24·9)	107 (25·7)	96 (24·9)	134 (28·9)	115 (24·1)	102 (25·1)
50–69	15 (3·5)	12 (2·7)	15 (3·6)	11 (2·6)	7 (1·8)	9 (1·9)	11 (2·3)	15 (3·7)
70+	16 (3·8)	9 (2·0)	17 (4·1)	15 (3·6)	4 (1·0)	2 (0·4)	4 (0·8)	4 (1·0)
Education attempted								
None	30 (7·0)	38 (8·5)	32 (7·7)	33 (7·9)	65 (16·9)	85 (18·3)	63 (13·2)	59 (14·5)
Primary	85 (20·0)	107 (24·0)	96 (23·0)	83 (19·9)	92 (23·9)	128 (27·6)	121 (25·4)	101 (24·9)
O-level	126 (29·6)	133 (29·9)	102 (24·5)	123 (29·5)	101 (26·2)	106 (22·8)	129 (27·0)	113 (27·8)
A-level	110 (25·8)	106 (23·8)	99 (23·7)	93 (22·3)	53 (13·8)	62 (13·4)	61 (12·8)	54 (13·3)
Tertiary or higher	75 (17·6)	59 (13·3)	88 (21·1)	85 (20·38)	74 (19·2)	83 (17·9)	103 (21·6)	79 (19·4)
Refused	0 (0·0)	2 (0·5)	0 (0·0)	0 (0·0)	-	-	-	-
Location								
Urban	220 (51·6)	247 (55·5)	199 (47·7)	236 (56·6)	218 (56·6)	240 (51·7)	278 (58·3)	234 (57·6)
Rural	204 (47·9)	197 (44·3)	218 (52·3)	181 (43·4)	167 (43·4)	224 (48·3)	199 (41·7)	172 (42·4)
Refused	2 (0·5)	1 (0·2)	0 (0·0)	0 (0·0)	-	-	-	-
Language								
Bangla	421 (98·8)	444 (99·8)	416 (99·8)	416 (99·8)	-	-	-	-
Luganda	-	-	-	-	220 (57·1)	287 (61·9)	295 (61·8)	264 (62·0)
Luo	-	-	-	-	17 (4·4)	30 (6·5)	20 (4·2)	18 (4·4)
Runyakitara	-	-	-	-	83 (21·6)	80 (17·2)	76 (15·9)	59 (14·5)
English	5 (1·2)	1 (0·2)	1 (0·2)	1 (0·2)	65 (16·9)	67 (14·4)	86 (18·0)	65 (16·0)

In Uganda, as in Bangladesh, the demographic characteristics were balanced across the four study arms (Table 2). More than two-thirds of the participants were males (74.2% - 77.3%) between the ages of 18–29 years (68.8% - 72.8%). The most common responses to highest level of education were primary-level (23.9% - 27.6%) and O-level (22.8% - 27.8%). Slightly more than half of the study population was in an urban location (51.7% - 58.3%). A majority of survey respondents elected to take the survey in Luganda (57.1% - 62.0%), followed by Runyakitara (14.5% - 21.6%), English (14.4% - 18.0%), and Luo (4.2% - 6.5%).

Table 3 examines the disposition codes of all calls placed across the four study arms. The estimated proportion of age-eligible unknowns, calculated using the known proportion methodology [38], was 79.0% (Bangladesh) and 90.7% (Uganda).

**Table 3:** Disposition codes by study arm in Bangladesh and Uganda, n (%).

Variable	Bangladesh				Uganda			
	Informational Intro		Motivational Intro		Informational Intro		Motivational Intro	
	Male Voice	Female Voice	Male Voice	Female Voice	Male Voice	Female Voice	Male Voice	Female Voice
Complete Interview (I)	426 (0.57)	445 (0.73)	417 (0.56)	417 (0.68)	385 (1.74)	464 (2.06)	477 (2.13)	406 (1.85)
Partial Interview (P)	184 (0.25)	107 (0.18)	190 (0.25)	164 (0.27)	95 (0.43)	126 (0.56)	143 (0.64)	104 (0.47)
Refusals (R)								
Refusal	214 (0.29)	194 (0.32)	346 (0.46)	276 (0.45)	73 (0.33)	97 (0.43)	109 (0.49)	65 (0.3)
Breaks-off	146 (0.19)	90 (0.15)	166 (0.22)	111 (0.18)	84 (0.38)	134 (0.6)	114 (0.51)	84 (0.38)
Unknown other (UO)	42068 (56.1)	34252 (56.09)	41846 (55.94)	34138 (55.95)	453 (2.05)	554 (2.46)	492 (2.2)	345 (1.57)
*e* Unknown *e*(UO)*	31593 (42.13)	25723 (42.13)	31426 (42.01)	25638 (42.02)	411 (1.86)	502 (2.23)	446 (1.99)	313 (1.43)
In-eligible (Underage)	358 (0.48)	252 (0.41)	415 (0.55)	267 (0.44)	72 (0.33)	85 (0.38)	85 (0.38)	62 (0.28)
Call did not connect†	-	-	-	-	17443 (78.91)	17443 (77.54)	17443 (77.87)	17442 (79.61)
Connected, but no language selection†	-	-	-	-	3091 (13.98)	3090 (13.74)	3090 (13.8)	3090 (14.1)

Non-contact (NC), Other (O), and Unknown household (UH) were all 0 for both countries.

*Estimated proportion of unknown cases that were age-eligible was 75.0% (Bangladesh) and 90.7% (Uganda, excluding call did not connect and no language selected).

† Evenly distributed to each study arm due to randomization occurring after language selection.

### Primary outcomes

Table 4 exhibits the survey rates for completed interviews across the study arms in Bangladesh and Uganda. In Bangladesh, the cooperation rates (i.e., the number of complete interviews divided by the sum of complete and partial interviews, refusals, and others) for the four study arms were: MI (43.9%); FI (53·2%); MM (37·3%); and FM (43·1%). Compared to the MI arm, the cooperation rate was significantly higher for the FI arm (RR: 1·21, 95% CI: 1·10–1·33, p<0.001) and significantly lower for the MM arm (RR: 0·85, 95% CI: 0·76–0·94, p = 0.002), with no detectable difference for the FM arm (RR: 0·98, 95% CI: 0·89–1·09, p = 0.71) in Bangladesh. In Uganda, the cooperation rates for the four study arms were: MI (56·5%); FI (56·6%); MM (37·3%); and FM (61·6%). With the MI arm as the reference, there was no detectable difference in the cooperation rate for the FI arm (RR: 0·94, 95% CI: 0·86–1·02, p = 0.13), the MM arm (RR: 0·98, 95% CI: 0·86–1·02, p = 0.13), and the FM arm (RR: 1·03, 95% CI: 0·93–1·11, p = 0.6) in Uganda.

**Table 4:** Survey rates by study arm in Bangladesh and Uganda (Shaded cells = p<0.05).

Survey rate	Bangladesh				Uganda			
	Informational Intro		Motivational Intro		Informational Intro		Motivational Intro	
	Male Voice (MI)	Female Voice (FI)	Male Voice (MM)	Female Voice (FM)	Male Voice (MI)	Female Voice (FI)	Male Voice (MM)	Female Voice (FM)
Contact Rate #2	43·0%	48·9%	50·0%	55·2%	60·8%	62·1%	65·4%	67·9%
Risk Ratio (95%CI)	*Ref*.	1·14 (1·06–1·22)	1·16 (1·09–1·24)	1·28 (1·20–1·37)	*Ref*.	1·02 (0·96–1·09)	1·07 (1·01–1·14)	1·12 (1·05–1·19)
p-value	*Ref*.	<0.001	<0·001	<0·001	*Ref*.	0·55	0·024	0·001
Response Rate #4	27·1%	32·3%	27·2%	33·1%	45·9%	44·6%	48·1%	52·5%
Risk Ratio (95%CI)	*Ref*.	1·19 (1·08–1·31)	1·00 (0·91–1·10)	1·22 (1·11–1·35)	*Ref*.	0·97 (0·89–1·06)	1·05 (0·96–1·14)	1·15 (1·05–1·25)
p-value	*Ref*.	<0·001	0·95	<0·001	*Ref*.	0·54	0·28	0·003
Refusal Rate #2	16·0%	16·6%	22·9%	22·1%	15·0%	17·5%	17·3%	15·4%
Risk Ratio (95%CI)	*Ref*.	1·04 (0·90–1·20)	1·43 (1·27–1·62)	1·38 (1·21–1·57)	*Ref*.	1·16 (0·97–1·40)	1·15 (0·96–1·39)	1·02 (0·83–1·26)
p-value	*Ref*.	0·58	<0·001	<0·001	*Ref*.	0·11	0·13	0·83
Cooperation Rate #1	43·9%	53·2%	37·3%	43·1%	60·4%	56·5%	56·6%	61·6%
Risk Ratio (95%CI)	*Ref*.	1·21 (1·10–1·33)	0·85 (0·76–0·94)	0·98 (0·89–1·09)	*Ref*.	0·94 (0·86–1·02)	0·98 (0·86–1·02)	1·03 (0·93–1·11)
p-value	*Ref*.	<0·001	0·002	0·71	*Ref*.	0·13	0·13	0·6

Abbreviations: CI: Confidence Interval MI: Male Informational

FI: Female Informational

MM: Male Motivational

FM: Female Motivational.

In Bangladesh, the response rates (i.e., number of complete interviews divided by the number of all possible eligible participants) of four study arms were: MI (27·1%); FI (32·3%); MM (27·2%); and FM (33·1%). With the MI arm as the reference, the response rate was significantly higher for the FI arm (RR: 1·19, 95% CI: 1·08–1·31, p<0.001) and the FM arm (RR: 1·22, 95% CI: 1·11–1·35, p<0.001), with no detectable difference for the MM arm (RR: 1·00, 95% CI: 0·91–1·10, p = 0.95) in Bangladesh. In Uganda, the response rates for the four study arms were: MI (45·9%); FI (44·6%); MM (48·1%); and FM (52·5%). With the MI arm as the reference, there was no detectable difference in the response rate for the FI arm (RR: 0·97, 95% CI: 0·89–1·06, p = 0.54) and the MM arm (RR: 1·05, 95% CI: 0·96–1·14, p = 0.28), and an increased response rate in the FM arm (RR: 1·15, 95% CI: 1·05–1·25, p = 0.003) in Uganda (Table 4).

### Secondary outcomes

Contact rates (indicating the proportion of persons reached by the survey) were 43.0%, 48.9%, 50.0%, and 55.2%, respectively in MI, FI, MM, and FM arms in Bangladesh; there was a significant increase in the contact rate in the FI (RR: 1·14, 95% CI: 1·06–1·22, p<0.001), MM (RR: 1·16, 95% CI: 1·09–1·24, p<0.001), and FM (RR: 1·28, 95% CI: 1·20–1·37, p<0.001) arms in Bangladesh compared to the MI arm. In Uganda, the Contact rates (indicating the proportion of persons reached by the survey) were 43.0%, 48.9%, 50.0%, and 55.2%, respectively in MI, FI, MM, and FM arms in Bangladesh; there was a significant increase in the contact rate in the FI (RR: 1·14, 95% CI: 1·06–1·22, p<0.001), MM (RR: 1·16, 95% CI: 1·09–1·24, p<0.001), and FM (RR: 1·28, 95% CI: 1·20–1·37, p<0.001) arms in Bangladesh compared to the MI arm. In Uganda, the contact rates across the four arms were 60·8%, 62·1%, 65·4%, and 67·9%, respectively in MI, FI, MM, and FM arms; compared to the MI arm, there was no difference in the contact rate in the FI arm (RR: 1·02, 95% CI: 0·96–1·09, p = 0.55), and an increase in the MM (RR: 1·07, 95% CI: 1·01–1·14, p = 0.024) and FM (RR: 1·12, 95% CI: 1·05–1·19, p = 0.001) arms (Table 4).

The refusal rates (indicating the proportion of breakoffs and refusals among those eligible to participate in the survey) were 16.0%, 16.6%, 22.9%, and 22.1%, respectively, across the four study arms (i.e., MI, FI, MM, and FM) in Bangladesh. With the MI arm as reference, there was no difference in the contact rate in the FI arm (RR: 1·04, 95% CI: 0·90–1·20, p<0.001), and an increase in the MM (RR: 1·43, 95% CI: 1·27–1·62, p<0.001) and FM arms (RR: 1·38, 95% CI: 1·21–1·57, p<0.00`). In Uganda, the refusal rates were 15.0%, 17·5%, 17·3%, and 15·4% in MI, FI, MM, and FM arms, respectively. With the MI arm as reference, there was no difference in the refusal rate in the FI arm (RR: 1·16, 95% CI: 0·97–1·40, p = 0.11), the MM arm (RR: 1·15, 95% CI: 0·96–1·39, p = 0.13), or the FM arm (RR: 1·02, 95% CI: 0·83–1·26, p = 0.83) (Table 4).

### Pooled analysis

Table 5 evaluates group samples to look for the effects of survey voice introduction in both countries. Using the pooled sample, in Bangladesh, the cooperation rates were: MV (40·4%), FV (47·8%), II (48·2%), and MI (40·0%), with higher cooperation rates seen in the arm using a female voice (RR: 1·18, 95% CI: 1·10–1·27, p<0.001) as compared to a male voice, and lower cooperation rates in the arm using a motivational introduction (RR: 0·83, 95% CI: 0·77–0·89, p<0.001) as compared to an informational one. In Uganda, the cooperation rates were: MV (58·2%), FV (58·8%), II (58·2%), and MI (58·8%); there was no difference in cooperation rates in the arm using a female voice (RR: 1·01, 95% CI: 0·95–1·07, p = 0.76) as compared to a male voice, or in the arm using a motivational introduction (RR: 1·01, 95% CI: 0·95–1·07, p = 0.76) as compared to an informational one.

In Bangladesh, the refusal rates were: MV (19·4%), FV (19·4%), II (16·3%), and MI (22·5%), with no difference in refusal rates in the arm using a female voice (RR: 1·00, 95% CI: 0·91–1·09, p = 0.95) as compared to a male voice, and a higher refusal rate in the arm using a motivational introduction (RR: 1·39, 95% CI: 1·27–1·52, p<0.001) as compared to an informational one. In Uganda, the refusal rates were: MV (16·3%), FV (16·6%), II (16·4%), and MI (16·5%), with no difference in refusal rates in the arm using a female voice (RR: 1·02, 95% CI: 0·89–1·16, p = 0.66) as compared to a male voice, or in the arm using a motivational introduction (RR: 1·01, 95% CI: 0·88–1·15, p = 0.94) as compared to an informational one.

In Bangladesh, the contact rates were: MV (46·5%), FV (52·1%), II (45·6%), and MI (52·3%); the contact rate was higher with the use of a female voice compared to a male voice (RR: 1.12, 95% CI: 1.07–1.17, p<0.001), and for the use of a motivational introduction (RR: 1.15, 95% CI: 1.10–1.20, p<0.001) compared to the use of an informational introduction. In Uganda, the contact rates were 63·4%, 64·5%, 61·5%, and 66·5% for MV, FV, II, and MI, respectively; it was not significantly different with the use of a female voice compared to a male voice (RR: 1.02, 95% CI: 0.98–1.06, p = 0.41) and was increased for the motivational introduction (RR: 1.08, 95% CI: 1.03–1.13, p<0.001) as compared to the informational introduction.

The refusal rates in the pooled sample in Bangladesh were: MV (19·4%), FV (19·4%), II (16·3%), and MI (22·5%); there was no difference in refusal rate when comparing female voice to male voice (RR: 1·00, 95% CI: 0·91–1·09), and an increase in refusal rate with motivational introduction as compared to informational introduction (RR: 1·39, 95% CI: 1·27–1·52, p<0.001). In Uganda, the refusal rates were 16·3%, 16·6%, 16·4%, and 16·5% respectively; there was no difference in refusal rates with use of a female voice compared to a male voice (RR: 1·02, 95%CI: 0·89–1·16, p = 0.78) or with the use of a motivational introduction as compared to an informational one (RR:1·01, 95% CI: 0·88–1·15, p = 0.94).

**Table 5:** Pooled survey rates when combining by survey voice and introduction in Bangladesh and Uganda.

Survey rate	Bangladesh				Uganda			
	Gender Effect (Pooling Intros)		Intro Effect (Pooling Genders)		Gender Effect (Pooling Intros)		Intro Effect (Pooling Genders)	
	Male (MV)	Female (FV)	Informational (II)	Motivational (MI)	Male (MV)	Female (FV)	Informational (II)	Motivational (MI)
Contact Rate #2	46·5%	52·1%	45·6%	52·3%	63·4%	64·5%	61·5%	66·5%
Risk Ratio (95%CI)	*Ref*.	1·12 (1·07–1·17)	*Ref*.	1·15 (1·10–1·20)	*Ref*.	1·02 (0·98–1·06)	*Ref*.	1·08 (1·03–1·13)
p-value	*Ref*.	<0·001	*Ref*.	<0·001	*Ref*.	0·41	*Ref*.	<0·001
Response Rate #4	27·1%	32·7%	29·3%	29·8%	47·1%	48·0%	45·2%	50·0%
Risk Ratio (95%CI)	*Ref*.	1·21 (1·13–1·29)	*Ref*.	1·02 (0·95–1·09)	*Ref*.	1·02 (0·96–1·08)	*Ref·*	1·11 (1·04–1·18)
p-value	*Ref*.	<0·0001	*Ref*.	0·66	*Ref*.	0·56	*Ref·*	0·001
Refusal Rate #2	19·4%	19·4%	16·3%	22·5%	16·3%	16·6%	16·4%	16·5%
Risk Ratio (95%CI)	*Ref*.	1·00 (0·91–1·09)	*Ref*.	1·39 (1·27–1·52)	*Ref*.	1·02 (0·89–1·16)	*Ref·*	1·01 (0·88–1·15)
p-value	*Ref*.	0·95	*Ref*.	<0·001	*Ref*.	0·78	*Ref·*	0·94
Cooperation Rate #1	40·4%	47·8%	48·2%	40·0%	58·2%	58·8%	58·2%	58·8%
Risk Ratio (95%CI)	*Ref*.	1·18 (1·10–1·27)	*Ref*.	0·83 (0·77–0·89)	*Ref*.	1·01 (0·95–1·07)	*Ref·*	1·01 (0·95–1·07)
p-value	*Ref*.	<0·001	*Ref*.	<0·001	*Ref*.	0·76	*Ref·*	0·76

Abbreviations: CI: Confidence interval

MV: Male voice

FV: Female voice

II: Informational Intro

MI: Motivational Intro.

(p<0.05 shaded).

### Sensitivity analyses

Sensitivity analyses were conducted to check for differences in respondent characteristics among complete and partial interviews as defined in the study, with no differences detected (S2 Table). Analyses were also conducted to look for differences in cooperation rate with the introduction of an interaction term: survey voice (S3 Table) and survey introduction (S4 Table), with none detected across demographic characteristics.

## Discussion

We conducted this IVR survey in Bangladesh and Uganda using RDD with participants randomized to one of four combinations of male or female voice and an informational or motivational introduction to test the effects of such combinations on survey performance metrices. Demographic characteristics were comparable across the study arms in both countries. The findings in Bangladesh suggest that the use of a female voice in mobile surveys may increase contact, cooperation, and cooperation. The findings of reduced cooperation rate and increased refusal rate when using a motivational introduction in Bangladesh are in contrast to the higher response rate in Uganda with a motivational introduction. This study adds significant knowledge to the growing body of literature investigating the impact of types of voice and introductory message on the survey performance metrices.

The positive impact of female voice on overall survey performance metrices both before and after pooling by introductions in Bangladesh indicates that using a female voice could reduce the cost of conducting IVR surveys in that country. However, there was a lack of such association in participation in Uganda. Prior research in IVR optimization strove to eliminate speaker-listener gender differentials as a way of minimizing potential adverse reactions from spouses or household members, such as a husband being suspicious if a wife takes a call from an unfamiliar male voice. Sensitivity to such local gender dynamics may improve participation [39].

In Uganda, while there were higher contact, response, and cooperation rates but lower refusal rates compared to Bangladesh. It is unclear if the shift seen in Bangladesh can be attributed to survey fatigue, distrust of motivational messaging, or other survey-related factors. This difference may also be attributable to mobile network issues or survey length, both of which deserve further exploration. We should note that the contact rate findings should be interpreted with caution as they may not represent a response to the study exposure (i.e., the choice of voice gender or type of introductory message)–contact rates are an indication of the number of people that picked up the phone to answer the RDD. It is also not surprising that the differences in survey rates by gender and nature of introduction appear to be highly country-specific, given stark cultural differences between Bangladesh and Uganda [40,41]. While gender roles remain fairly traditional in both countries, there are differences within each setting that may contribute to the findings. For instance, Uganda has a roughly 68% public labor force participation rate among women, while this rate is 36% in Bangladesh [42]. With fewer women participating in the public workforce in Bangladesh, mixed gender interactions may be viewed as less appropriate. Young females (aged 15–24) in both countries, however, are 80–85% literate, revealing a much smaller difference among younger women. Despite reportedly high rates of overall mobile ownership in both countries (>65%), the 2018 GSMA Mobile Gender Gap report found a 33% gender gap in phone ownership in Bangladesh, while 2015 statistics suggest this gap is ~10% smaller in Uganda–with 77% of men owning phones vs. only 54% of women [42,43].

Given the increasing numbers of unsolicited commercial phone calls and text messages sent to mobile users in all countries [10,14], strategies to improve engagement with calls made for a collection of publicly useful data (a public good) are necessary–and may need to include ways to differentiate these calls, in addition to methods to increase already low rates of successful engagement, however, measured. Previous research has shown that survey respondents respond well to “linguistic congruence”, where the speaker in the recorded messages is from the same cultural or ethnic group [44]. In these studies, this was always the case, but it is important to note that in addition to cultural congruence, an understanding of how female or male speakers are seen as ‘trustworthy’ in a particular culture may be additionally important.

Ultimately, however, policy actions taken to reduce the background ‘noise’ represented by receiving many unsolicited advertising calls, texts, and IVR messages are likely to improve respondents willingness to engage with mobile surveys [5,14,45]. Leeper recently posited that phone survey non-response represents a “common pool resource” problem–which, similar to unregulated and unrestricted fishing can rapidly deplete the proverbial “ocean” of potential respondents [46]. It may not be plausible to consider regulating or limiting the number of phone surveys launched across the public and private sector, so efforts to motivate putative respondents to participate will need to continue.

Future qualitative research exploring the drivers of varied responses to different gendered voices and kinds of introductory messages might help with understanding the drivers of survey responses in different contexts. Other nuances that “hook” the call recipient, such as playfulness or the use of respected leaders or celebrities, have been tested in other advertising and media channels and might lend their success to this survey space as well [25,47].

The limitations of the present study also warrant discussion. In Uganda, for logistical reasons, four of the local languages and English were used to deliver the survey. Due to limited information about respondents’ survey-taking behavior, the reasons why a particular approach did or did not work in each context are difficult to glean. This may also lead to challenges with external validity, as these approaches of survey voice and survey introduction may have different effects in different contexts. There may also be confounding introduced by pooling across survey voice and survey introduction when examining survey outcomes.

Despite these limitations, this study has several notable strengths. It seeks to answer important questions about motivating survey respondents to engage in mobile-phone or phone surveys, writ large. This study uses a randomized, controlled design, and leverages a standardized questionnaire adapted from the WHO STEPwise survey questions [48], dialing participant numbers through random digit dialing. The study is adequately powered to draw inferences about the impact of these strategies (survey voice gender and survey introduction type) on survey success. These sorts of explorations into survey participation, particularly gendered effects, will help to reduce the gendered data gap in health and other data.

## Conclusion

This study showed relatively better performance of using a female voice or a motivational introduction in Bangladesh. Different results were observed in Uganda, demonstrating the need for a better understanding of the impact of gendered voice and introductory message by cultural context on survey performance metrices. Further exploration of such nuances is crucial for not only improving survey participation in LMICs but also for reducing gendered data gaps in these survey modalities.

## Supporting information

S1 Checklist CONSORT 2010 checklist of information to include when reporting a randomised trial*. (DOC)

S1 Table Script of motivational and information intro. (DOCX)

S2 Table Demographics of complete interviews and partial interviews in Bangladesh and Uganda, n (%). (DOCX)

S3 Table Sub-group analyses of cooperation rates for survey voice in Bangladesh and Uganda. (DOCX)

S4 Table Sub-group analyses of cooperation rates for survey introduction in Bangladesh and Uganda. (DOCX)

S1 File (DOCX)
