# Supplementary material for: Improving success of non-communicable diseases mobile phone surveys: Results of two randomized trials testing interviewer gender and message valence in Bangladesh and Uganda
Source: PLoS One. 2023 May 24;18(5):e0285155. doi: 10.1371/journal.pone.0285155 (PMC10208499; doi:10.1371/journal.pone.0285155)
Supplement: S1 Table — (DOCX) [file pone.0285155.s002.docx]

**S1 Table: Script of motivational and information intro**

| Bangladesh | Informational | Thank you. This interview will take about 20 minutes of your time. All information you provide will be kept confidential and private. To answer each question, I will ask you to press a certain number, for example, press 1 for YES, and 3 for NO or to answer a question with a number such as 12, tap 1 and 2. At any time, you can repeat the question by pressing the star button. If you are unwilling to answer any question, you can press 0.  As a token of appreciation, we will send you 50 Taka of talktime for completing the survey  To listen to the questions clearly and to respond easily, you may turn on your mobile set speaker. |
| --- | --- | --- |
|  | Motivational | Your answers could help improve healthcare in communities across our country.  For completing the survey, we’ll send you 50 Taka talk time as a token of our appreciation.  You’ll be able to answer all of the questions by pressing a number on your phone.  And all of your answers will remain strictly confidential. Please take a few minutes to help make our country healthier and happier. |
| Uganda | Informational | Hello, we are conducting a heath survey from Makerere University. This interview will take no more than 20 minutes of your time. Any information you share will be kept confidential and private. To answer each question, I will ask you to press a number - for example, 1 for YES, and 3 for NO, or to answer a question with a number - like 12 or 22. You can hear a question again at any time by pressing the STAR button - located to the left of the ZERO at the bottom of your phone. As a token of appreciation, we will send you 5000 Shillings of airtime for completing the survey |
|  | Motivational | “Hello, we are conducting a heath survey from Makerere University. Your answers could help improve healthcare in communities across our country. For completing the survey, we’ll send you 5000 Shillings of airtime as a token of our appreciation. You’ll be able to answer all of the questions by pressing a number on your phone. And all of your answers will remain strictly confidential. Please take a few minutes to help make our country healthier and happier.” |
