# Supplementary material for: Improving success of non-communicable diseases mobile phone surveys: Results of two randomized trials testing interviewer gender and message valence in Bangladesh and Uganda
Source: PLoS One. 2023 May 24;18(5):e0285155. doi: 10.1371/journal.pone.0285155 (PMC10208499; doi:10.1371/journal.pone.0285155)
Supplement: S2 Table — (DOCX) [file pone.0285155.s003.docx]

# **S2 Table. Demographics of complete interviews and partial interviews in Bangladesh and Uganda, n (%)**

| Variables | **Bangladesh** | | | **Uganda** | | |
| --- | --- | --- | --- | --- | --- | --- |
|  | Complete Interviews (n=1705) | Partial Interviews (n=645) | p-value | Complete Interviews  (n =1732) | Partial Interviews (n=468) | p-value |
| Gender |  |  |  |  |  |  |
| Male | 1476 (86.6) | 540 (83.7) | 0.21 | 1319 (76.2) | 335 (71.6) |  |
| Female | 215 (12.6) | 98 (15.2) |  | 413 (23.9) | 98 (28.4) | **0.042** |
| Transgender | 14 (0.8) | 7 (1.1) |  | - | - |  |
| Age group (years) |  |  |  |  |  |  |
| 18-29 | 1146 (67.2) | 429 (66.6) |  | 1229 (71.0) | 321 (68.6) |  |
| 30-49 | 449 (26.3) | 159 (24.7) | 0.09 | 447 (25.8) | 128 (27.4) | 0.30 |
| 50-69 | 53 (3.1) | 21 (3.3) |  | 42 (2.4) | 11 (2.4) |  |
| 70+ | 57 (3.3) | 36 (5.6) |  | 14 (0.8) | 8 (1.7) |  |
| Education attempted |  |  |  |  |  |  |
| None | 133 (7.8) | 59 (9.2) |  | 272 (15.7) | 78 (16.7) |  |
| Primary | 371 (21.8) | 139 (21.6) |  | 442 (25.5) | 136 (29.1) |  |
| O-level | 485 (28.4) | 201 (31.2) | 0.13 | 449 (25.9) | 106 (22.7) | 0.39 |
| A-level | 408 (23.9) | 150 (23.3) |  | 230 (13.3) | 64 (13.7) |  |
| Tertiary or higher | 307 (18.0) | 93 (14.4) |  | 329 (19.6) | 84 (18.0) |  |
| Refused | 2 (0.1) | 3 (0.5) |  | - | - |  |
| Location |  |  |  |  |  |  |
| Urban | 902 (52.9) | 354 (55.9) |  | 970 (56.0) | 273 (59.3) |  |
| Rural | 800 (46.9) | 286 (44.3) | 0.32 | 762 (44.0) | 195 (41.7) | 0.37 |
| Refused | 3 (0.2) | 5 (0.8) |  |  |  |  |
| Language |  |  |  |  |  |  |
| Bangla | 1697 (99.5) | 640 (99.2) | 0.37 | - | - |  |
| Luganda | - | - |  | 1066 (61.6) | 296 (63.3) | 0.72 |
| Luo | - | - |  | 85 (4.9) | 21 (4.5) |  |
| Runyakitara | - | - |  | 298 (17.2) | 71 (15.2) |  |
| English | 8 (0.5) | 5 (0.8) |  | 283 (16.3) | 80 (17.1) |  |
