# Supplementary material for: Improving success of non-communicable diseases mobile phone surveys: Results of two randomized trials testing interviewer gender and message valence in Bangladesh and Uganda
Source: PLoS One. 2023 May 24;18(5):e0285155. doi: 10.1371/journal.pone.0285155 (PMC10208499; doi:10.1371/journal.pone.0285155)
Supplement: S3 Table — (DOCX) [file pone.0285155.s004.docx]

**S3 Table. Sub-group analyses of cooperation rates for survey voice in Bangladesh and Uganda.**

|  | **Bangladesh** | | | | **Uganda** | | | |
| --- | --- | --- | --- | --- | --- | --- | --- | --- |
|  | Male Voice, n/N (%) | Female Voice, n/N (%) | Stratum-specific RR* | p-val† | Male Voice, n/N (%) | Female Voice, n/N (%) | Stratum-specific RR* | p-val† |
| Age (years) |  |  |  |  |  |  |  |  |
| 18-29 | 561/1328 (42.2) | 585/1132 (51.7) | 1.22  (1.12 – 1.33) | 0.28 | 625/1229 (50.9) | 604/1229 (49.1) | 1.00  (0.93 - 1.08) | 0.66 |
| 30-99 | 282/761 (37.1) | 277/672 (41.2) | 1.12  (0.98 – 1.27) |  | 237/503 (47.1) | 266/503 (52.9) | 1.03  (0.92 - 1.16) |  |
| Gender |  |  |  |  |  |  |  |  |
| Male | 731/1282 (57.0) | 770/1167 (66.0) | 1.16  (1.09 – 1.23) | 0.93 | 647/1319 (49.1) | 672/1319 (50.9) | 1.00  (0.94 - 1.07) | 0.59 |
| Female | 119/218 (54.6) | 104/166 (62.7) | 1.15  (0.97 – 1.36) |  | 215/413 (52.1) | 198/413 (47.9) | 0.97  (0.86 - 1.09) |  |
| Location |  |  |  |  |  |  |  |  |
| Urban | 431/771 (55.9) | 493/753 (65.5) | 1.17  (1.08 – 1.27) | 0.70 | 496/970 (51.1) | 474/970 (48.9) | 0.97 (0.9 - 1.04) | 0.30 |
| Rural | 427/709 (60.2) | 384/557 (68.9) | 1.14  (1.05 – 1.24) |  | 366/762 (48) | 396/762 (52) | 1.03  (0.95 - 1.11) |  |
| Education |  |  |  |  |  |  |  |  |
| ≤Primary | 244/452 (54.0) | 262/388 (67.5) | 1.25  (1.12 – 1.40) | 0.098 | 341/714 (47.8) | 373/714 (52.2) | 1.04  (0.95 - 1.13) | 0.20 |
| ≥Secondary | 616/1008 (61.1) | 614/895 (68.6) | 1.12  (1.05 – 1.20) |  | 521/1018 (51.2) | 497/1018 (48.8) | 0.97  (0.91 - 1.03) |  |

* Male voice is reference

† p values obtained from an interaction term between study arm and demographic characteristic
