# Supplementary material for: Improving success of non-communicable diseases mobile phone surveys: Results of two randomized trials testing interviewer gender and message valence in Bangladesh and Uganda
Source: PLoS One. 2023 May 24;18(5):e0285155. doi: 10.1371/journal.pone.0285155 (PMC10208499; doi:10.1371/journal.pone.0285155)
Supplement: S4 Table — (DOCX) [file pone.0285155.s005.docx]

**S4 Table: Sub-group analyses of cooperation rates for survey introduction in Bangladesh and Uganda.**

|  | **Bangladesh** | | | | **Uganda** | | | |
| --- | --- | --- | --- | --- | --- | --- | --- | --- |
|  | Male Voice, n/N (%) | Female Voice, n/N (%) | Stratum-specific RR* (95% CI) | p valueⱡ | Male Voice, n/N (%) | Female Voice, n/N (%) | Stratum-specific RR* (95% CI) | p valueⱡ |
| Age (years) |  |  |  |  |  |  |  |  |
| 18-29 | 581/1135 (51.2) | 565/1325 (42.6) | 0.83  (0.77 – 0.91) | 0.80 | 597/1229 (48.6) | 632/1229 (51.4) | 1.01  (0.94 - 1.09) | 0.89 |
| 30-99 | 290/671 (43.2) | 269/762 (35.3) | 0.82  (0.72 – 0.93) |  | 252/503 (50.1) | 251/503 (49.9) | 1.00 (0.89 - 1.13) |  |
| Gender |  |  |  |  |  |  |  |  |
| Male | 750/1172 (64.0) | 726/1233 (58.9) | 0.92  (0.86 – 0.98) | 0.57 | 651/1319 (49.4) | 668/1319 (50.6) | 0.98  (0.92 - 1.04) | 0.30 |
| Female | 114/195 (58.5) | 101/178 (56.7) | 0.97  (0.82 – 1.16) |  | 198/413 (47.9) | 215/413 (52.1) | 1.05  (0.94 - 1.18) |  |
| Location |  |  |  |  |  |  |  |  |
| Urban | 467/747 (62.5) | 435/740 (58.8) | 0.94  (0.87 – 1.02) | 0.69 | 458/970 (47.2) | 512/970 (52.8) | 1.03  (0.95 - 1.1) | 0.24 |
| Rural | 401/599 (67.0) | 399/649 (61.5) | 0.92  (0.85 – 1.00) |  | 391/762 (51.3) | 371/762 (48.7) | 0.96  (0.89 - 1.04) |  |
| Education |  |  |  |  |  |  |  |  |
| ≤Primary | 260/418 (62.2) | 244/415 (58.1) | 0.95  (0.85 – 1.06) | 0.68 | 370/714 (51.8) | 344/714 (48.2) | 1  (0.92 - 1.09) | 0.71 |
| ≥Secondary | 609/904 (67.4) | 590/952 (61.8) | 0.92  (0.86 – 0.98) |  | 479/1018 (47.1) | 539/1018 (52.9) | 0.98  (0.92 - 1.05) |  |

* Male voice is reference

† p values obtained from an interaction term between study arm and demographic characteristic
