# Supplementary material for: Improving success of non-communicable diseases mobile phone surveys: Results of two randomized trials testing interviewer gender and message valence in Bangladesh and Uganda
Source: PLoS One. 2023 May 24;18(5):e0285155. doi: 10.1371/journal.pone.0285155 (PMC10208499; doi:10.1371/journal.pone.0285155)
Supplement: S1 File — (DOCX) [file pone.0285155.s006.docx]

**JHSPH IRB Research Plan for New Data Collection**

**PI Name:** Adnan Hyder

**Study Title:** Evaluation of mechanisms to improve mobile phone surveys- Optimization Phase

**IRB No.:** IRB 00007318

**PI Version No./Date: Version**  September 12, 2016

**Aims of the Study:**

***Describe the aims/objectives of the research and/or the project’s research questions or hypotheses.***

The Bloomberg Philanthropies Data for Health initiative aims to strengthen the collection and use of critical public health information. One of the components of the initiative seeks to explore innovative approaches to non-communicable disease (NCD) surveillance, including the use of mobile phone surveys for NCDs, in 10 countries. The main objectives of the component are to assess the feasibility, quality, and validity of NCD mobile phone surveys.

This protocol builds onto currently approved protocols for the *formative phase* (JHU IRB No: 0000709); where the aim of the formative phase is to contextualize the use of mobile phone surveys on an *Interactive Voice Response* (IVR) platform for usability and optimal performance in participating countries. This IRB submission describes the **optimization phase** of the NCD risk factor mobile phone survey.

This protocol’s overall goal is to answer key questions on how to improve IVR survey participation and how mobile phone survey modalities (IVR versus Computer Assisted Telephone Interview [CATI]) differ on key survey performance metrics. With IVR surveys, respondents interact with a pre-programmed database which contains both questions, and a series of pre-set answers to the questions - linked to a specific numeric key, or numeric response on a touch-tone phone keypad (e.g. “Press 1 for Yes”). CATI surveys employ call centers where human interviewers follow a script provided by a software program to survey participants. These results will inform future implementation of mobile phone surveys in low- and middle-income countries (LMICs). Through a set of seven rapid, “*microtrials”*, the protocol seeks to:

Primary Objectives:

1. Evaluate the impact of *incentives* on response, completion, and refusal rates of an IVR-administered NCD risk factor survey.
2. Assess the effect of different IVR survey *introduction modalities* on response, completion, and refusal rates of an IVR-administered NCD risk factor survey.
3. Examine the differences in survey metrics, such as representativeness, completeness, and response rate, between random digit dialing (RDD) approaches and existing sources e.g., Mobile Network Operators (MNO).
4. Evaluate the differences in survey metrics between an IVR Survey and a Computer Assisted Telephone Interview (CATI).

**II. Background and Rationale:**

***Explain why this study is being done. Summarize briefly what is already known about the issue and reference previously published research, if relevant.***

Non communicable diseases (NCDs) are a growing burden in lower- and middle-income countries (LMICs).^1-3^ Approximately 28 million, or 75%, of all NCD deaths occur in lower and middle income countries.^4^ Physical inactivity, poor diet, alcohol use, and smoking are the four most common risk factors associated with NCDs.^5^

To assess the burden and prevalence of NCDs and risk factors, the World Health Organization (WHO) supports countries with the implementation and analysis of the STEPS survey.^6^ The STEPS survey contains three steps, or components, of NCD surveillance: (1) self-reported risk factor questionnaire, (2) physical measurement, and (3) biochemical measurement. The risk factor questionnaire contains questions on tobacco use, alcohol consumption, diet, physical activity, history of NCDs, and lifestyle advice and it is administered face-to-face (F2F) with the respondent. The remaining two steps do not employ questionnaires, but rather take physical measurements (such as height and weight) and biochemical measurements (blood glucose).

Although the World Health Organization supports a household survey (STEPS) to assess national level NCD risk factor prevalence, the high costs and time commitments of conducting such face-to-face surveys means that they are only conducted approximately every 5-10 years. A more frequent collection of NCD risk factors could address gaps in NCD surveillance, assist policy makers by directing resources to NCD prevention programs, and also allow for a more timely assessment of such policies and programs, while also complementing existing data sources with additional population-level insights.

To address the high costs and time requirements associated with household surveys, higher income countries have developed and employed telephone and mobile phone surveys to collect population-level estimates of health and demographics.^7-9^ Concomitant with the global increase in mobile phone ownership and access^10^ opportunities exist to leverage mobile-health technologies and communication channels to revolutionize the current methods of data collection in low- and middle-income countries (LMIC)--- face-to-face interviews at respondent’s household--- by now interviewing respondents over their own personal mobile phone through the use of short message service (SMS; or ‘text message’), interactive voice response (IVR), and computer assisted telephone interviews (CATI) survey modalities; collectively called mobile phone surveys (MPS). This protocol focuses on mechanisms to optimize the performance of IVR surveys conducted in LMICs and to compare how different survey modalities (i.e. CATI versus IVR) affect the survey metrics.

As MPS in LMIC settings are relatively new, there is a dearth of scientific evidence on factors that affect MPS’s response, completion, and attrition rates and corresponding mechanisms to improve them in this context. The optimization of surveys with respect to incentives, survey introductions, and sampling frame (i.e. source of mobile phone number) to improve these survey metrics strengthens scientific rigor and the rationale for future nationally administered MPS, and has the potential to create significant cost-savings by enhancing survey response and completion rates.

**III. Study Design**

A. ***Provide an overview of your study design and methods. The study design must relate to your stated aims/objectives. Details will be requested later.***

The following objectives will be conducted in four of the following six countries: Uganda, Bangladesh, Tanzania, Kenya, Lebanon, and Ghana. The Bloomberg Philanthropies Data for Health Initiative (under which this work falls), has secured high level government agreements for Tanzania, Bangladesh, and Ghana to date. The four objectives detail a series of seven microtrials. Microtrials will be rapidly deployed, randomized survey deployments of limited sample sizes, sufficient to detect a consequential difference, as described below in Table 2 and will be sent through random digit dialing (RDD). RDD uses the mobile network operator identifying digit sequence as the base (e.g.all of MNO X’s phone numbers start with either 072, 077, or 079) and randomly generates the remaining digits to create a mobile phone number. A built-in randomization schema will randomly fork each dialed participant to one of the arms of the microtrial. To achieve these objectives, JHSPH will partner with in-country research institutes and government bodies. The countries will lead the research activities with support from JHSPH. JHSPH investigators will have no contact with research participants. VOTOmobile, a company that specializes in delivering IVR surveys globally, will be contracted to deliver the mobile phone surveys. De-identified data will be jointly shared with JHSPH and in-country partner investigators.

**Objective 1: Evaluation of the impact of incentives.** We will conduct three *microtrials* in order to assess the effect of providing airtime incentives on contact, response, completion, refusal, and attrition rates of IVR surveys. Prior to the first *microtrial*, we will conduct a baseline IVR survey to establish baseline completion rates and to finalize the questionnaire (see more details below). *Micro-trial #1* will assess whether the amount of incentive (e.g. 1 USD vs 2 USD) affects the survey metrics listed above. This incentive will be given to individuals who complete the IVR survey. *Micro-trial #2* will examine the effect of the incentive delivery’s timing (e.g. providing an incentive at the start of the survey versus at the end). The *third* micro-trial will assess the effect of the incentive structure by comparing survey metrics in those who receive a fixed incentive amount versus those who receive a ‘lottery’ incentive, where a lottery incentive is larger but only given to a small percentage of respondents. The contact, response, completion, refusal, and attrition rates will be calculated overall and stratified by key demographics.

**Objective 2: Evaluation of the impact of the survey’s introduction.** We will conduct two *microtrials* in order to assess the effect of the survey’s introduction on key survey metrics. *Micro-trial #3* will examine the introduction content (motivational vs informational) and survey’s voice (male vs female). *Micro-trial #4* will examine the effect of the introduction’s modality by observing differences in survey metrics in those who have a human operator provide the introduction, versus those who receive an IVR introduction. The contact, response, completion, refusal, and attrition rates will be calculated overall and stratified by key demographics.

**Objective 3: Evaluation of the impact of the survey’s sampling frame.**

The five *microtrials* presented in Objectives 1 and 2 will be sent to phone numbers that were randomly generated (i.e. a random digit dial [RDD] approach). Objective 3’s *microtrial* will send IVR surveys to a list of de-identified phone numbers that were provided by a mobile network operator (MNO; i.e. a cell phone company) and/or which were collected from a previous household survey where consent was collected. We will compare the demographic representativeness, response rate, completion rate, and cost from the IVR survey sent using MNO provided phone numbers with the IVR surveys from Objectives 1 and 2 in order to assess the effect of the sampling frame on these key survey metrics.

**Objective 4: Evaluation of the impact of the survey’s modality.**

We will conduct one *microtrial* in order to assess the effect of the survey’s modality (IVR versus CATI) on key survey metrics as described above and to assess inter-modal reliability. A group of participants will receive an IVR survey first, followed by a CATI survey 7 days later. A second group of participants will receive a CATI survey first followed by an IVR survey 7 days later. Whether a participant receives IVR or CATI first will be determined at random, blocking to ensure balance between IVR and CATI participation.

*B.* ***Provide a sample size and a justification as to how you arrived at that number. If you use screening procedures to arrive at a final sample a table may be helpful.***

A description of sample size calculations is provided below in the text and is accompanied with a table.

Baseline Survey: We selected 100 participants with complete surveys as a sample size because we think this a sufficient size sample to ascertain that the survey, incentive, and text message delivery mechanisms are well functioning. Adjusting our sample size to account for a baseline completion percentage of 30% (estimate provided by VOTOmobile), we anticipate to consent and enroll 334 participants per country; 1336 participants in four countries.

Microtrial 1-6:

Sample size calculations will be updated with country-specific completion percentages obtained from the baseline survey.

Assuming a baseline survey completion percentage of 30%, in order to detect an absolute 10% difference in survey completion between two study arms at an alpha of 0.05 and power of 80%, it is calculated that 376 individuals who have completed an IVR survey will be needed in each study arm for each microtrial. With a completion percentage of 30%, we calculated that 1254 participants will be required to consent to the survey per study arm. With 16 study arms in these 6 micro trials, 20,064 participants will be enrolled per country; 80,256 participants in four countries.

Microtrial 7:

This sample size is calculated based on the kappa statistic. With assumption of kappa= 0.75, a margin of error of 0.05%, an alpha of 0.05, and the proportion of positive responses=0.3, 405 participants who have completed the survey per study arm are needed. Adjusting for a 20% loss to follow-up from the first and second mobile phone survey and a 30% baseline completion percentage, 1688 participants who consented will be enrolled per arm and per country, for a total of 3376 participants across the two arms per country. With two arms and 4 countries, a total of 13,504 participants who provide consent across the four studies are needed.

Total Sample Size

For the baseline survey and seven microtrials, in each country, a total of 23,773 individuals who have consented to an MPS survey will be needed to fulfill a sample size of 6926 individuals who completed an MPS survey per country. As this protocol is being implemented in four countries, the overall sample size is 95,092 individuals who have consented to an MPS survey and 27,704 who completed a MPS survey

**Table 1.** Number of participants who consent to and complete a mobile phone survey per country

|  | **Col. A** | **Col. B** | **Col. C^*^**  [(Col B*100)/0.30] | **Col. D**  [Col A*Col B] | **Col. E**  [Col A*Col C] |
| --- | --- | --- | --- | --- | --- |
|  | # of study arms | N participants who completed survey per study arm | N participants who consented per study arm | Total N participants who completed survey | Total N participants who consented |
| **Objective 1** |  |  |  |  |  |
| *Baseline* | 1 | 100 | 334 | 100 | 334 |
| *Microtrial #1* | 3 | 376 | 1254 | 1128 | 3762 |
| *Microtrial #2* | 3 | 376 | 1254 | 1128 | 3762 |
| *Microtrial #3*** | 3 | 376 | 1254 | 1128 | 3762 |
| **Objective 2** |  |  |  |  |  |
| *Microtrial #4* | 4 | 376 | 1254 | 1504 | 5016 |
| *Microtrial #5* | 2 | 376 | 1254 | 752 | 2508 |
| **Objective 3** |  |  |  |  |  |
| *Microtrial #6* | 1 | 376 | 1254 | 376 | 1254 |
| **Objective 4** |  |  |  |  |  |
| *Microtrial #7* | 2 | 405 | 1688 | 810 | 3376 |
| **Total** |  |  |  | 6926 | 23,773 |

* For microtrial 7, we adjust for a 20% loss to follow-up from the first and second mobile phone survey.

In order to better understand reasons for refusal and survey attrition, to assess the usability of the IVR platform and receive feedback on the incentives and survey introductions, we will call a sub-sample of participants from *microtrials #1-5 and #7* and administer a short survey over the mobile phone. For each of these seven *microtrials*, we will call back a random sample of 30 individuals who did not complete the survey, 30 individuals who refused to consent to the survey, and 30 individuals who were identified as non-responders (i.e. did not initiate the survey); representing a total of 540 individuals in *microtrials* *#1-5, and 6,* per country.

**Fig 1**.Survey outcomes and number of participants who will complete a follow-up phone call

**
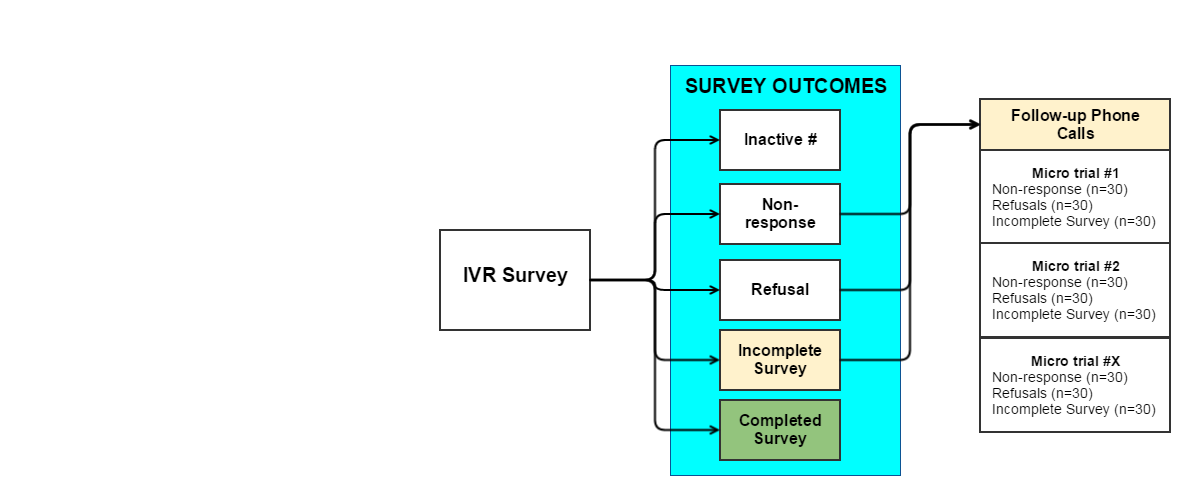
**

**IV. Participants**

***Describe the study participants and the population from which they will be drawn. Specify the inclusion and exclusion criteria. If you plan to include children, note their ages and whether you will include children in foster care. Note if the participants are particularly vulnerable in terms of cognitive limitations, education, legal migration status, incarceration, poverty, or some combination of factors.***

The protocol’s activities will be conducted in four of the following countries: Bangladesh, Lebanon, Kenya, Uganda, Tanzania, and Ghana. The PI and Co-I’s are actively engaging academic and research institutes from each of these six countries in order to secure their partnership.

Objectives 1,2 and 4

For *microtrials #1-5* from Objectives 1 and 2 and *microtrial #7* from Objective 4, potential participants will be randomly selected to answer an IVR survey (or CATI for *microtrial #7)*  through random digit dialing (RDD). Using the mobile network operator identifying digit sequence as the base (e.g.all of MNO X’s phone numbers start with either 072, 077, or 079) the remaining digits will be randomly generated to create a random sample of mobile phone numbers to which IVR surveys will be delivered. Numbers will then be dialed by computer software, connected to phone lines, until a human respondent answers the call.

Upon connecting to an active line, participants will be asked to select the language in which they would like to answer the survey. After hearing a brief introduction, participants will be asked to enter their age. Participants will be excluded from the survey if they report being less than 18 years old. There are no other inclusion or exclusion criteria for *microtrials* #1-5.

Objective 3

*Microtrial* #6 relies on phone numbers provided by an MNO or from a household survey where participants have consented to release their phone number for other studies. Participants in *microtrial* #6 will answer the same questionnaire as participants in *microtrials* # 1-5 and will similarly be screened for exclusion based on age---where those who report being less than 18 years old will be excluded.

**V. Study Procedures**

A. Recruitment Process:

1. ***Describe how you will identify, approach, and inform potential participants about your study. Include details about who will perform these activities and what their qualifications are.***

For all objectives, there will be no face-to-face or “in-person” interactions between study team members and research participants. We will work closely with in-country collaborators to obtain necessary national approvals for the study, including for the recruitment strategies outlined below.

For objectives 1, 2, and 4 the sampling of participants will be done using RDD. RDD is a method often used to select individuals to participate in a telephone survey through computer-facilitated random generation and dialing of digits that may comprise active telephone numbers. For objective 3, IVR surveys will be sent to a random sample of de-identified mobile phone numbers that were provided by a mobile network operator (MNO; i.e. a cell phone company or numbers list vendor) and/or which were collected from a previous household survey, where permission for re-contact was provided.

For all objectives, potential respondents will first receive a single SMS notification indicating that they will be sent a mobile phone survey shortly. The SMS will contain brief information on the survey’s purpose, the expected time commitment, and the survey’s sponsoring agency. An IVR survey will then be sent once to the same randomly generated mobile phone number where participants will be able to consent to the survey or refuse their participation (or CATI for microtrial #7). SMS notifications and IVR surveys will be sent by a mobile phone survey provider (e.g., VOTOmobile, a company that specializes in delivering IVR surveys and deploying RDD approaches globally) and who will be sub-contracted by investigators from JHU. For microtrials that contain a call center (*microtrials #5 and 7),* operators will be trained in human subjects research by the in-country partner. Training will include procedures on recruitment, consent, and interviewing techniques.

Once a respondent is contacted or participates in one of the proposed microtrial, they will be ineligible for participation in future microtrials. In each *microtrial*, there will be a single random selection of mobile phone numbers without replacement. However, since some participants own multiple mobile phones and phone numbers, it is possible that some participants may be randomly selected to participate in more than one microtrial.

*2.* ***Address any privacy issues associated with recruitment. If recruitment itself may put potential participants at risk (if study topic is sensitive, or study population may be stigmatized), explain how you will minimize these risks.***

Privacy issues associated with recruitment are anticipated to be minimal and similar to those associated with other face-to-face, phone or internet-based surveys. Participants will be sent an SMS alerting them of an upcoming survey and will then receive a phone call asking their willingness to respond to survey questions using their personal mobile phones. The incoming phone number will be the same for the text message and the phone call. Anyone not willing to complete the survey may choose to: 1) not answer the phone, 2) hang up any time after answering the phone, or 3) indicate their desire not to complete the survey by pressing a specified digit. Other individuals associated with or in proximity of respondents are unlikely to know that respondents are being recruited for the study unless the responded chooses to make this known.

B. Consent Process:

1. ***Describe the following details about obtaining informed consent from study participants. If a screening process precedes study enrollment, also describe the consent for screening.***

We are requesting a modification to written signed informed consent due to the nature of the research activities. Potential participants are unable to provide written informed consent as there is no face-to-face interaction. Moreover, as is common practice for brief IVR and CATI surveys, an abbreviated standardized verbal disclosure of key study information noting the voluntary nature of the survey will precede any data collection. After a brief introduction and opportunity for potential respondents to select their preferred language, potential participants will be read a brief description of the study and expected time burden in their language of choice. Potential participants will then be asked to “Press 1” if they would like to continue with the study. Individuals who do not “Press 1” will have the survey terminated. For participants in microtrial #5 and #7 where CATI surveys are conducted, the human operator will not proceed without a voluntary affirmative statement indicating the individual’s willingness to participate after he/she hears about the study.

*2.* ***Identify the countries where the research will take place, and the languages that will be used for the consent process.***

This study will be conducted in four of the following countries: Bangladesh, Lebanon, Kenya, Tanzania, Uganda, and Ghana. The consent/disclosure statement in the IVR survey will be made available in the most common languages for that study area. The statement will be translated into local languages and back-translated into English to ensure accuracy.

C. Study Implementation:

*1.* ***Describe the procedures that participants will undergo. If complex, insert a table below to help the reviewer navigate.***

**Study set-up**

Prior to enrollment and commencement of study, the NCD risk factor survey will be uploaded onto the web-based VOTOmobile IVR platform (See Appendix 1). VOTOmobile is a program that allows one to send audio recorded IVR surveys. The IVR surveys will be pre-tested prior to study commencement to identify and resolve any technical glitches, e.g. errors in skip patterns.

**Questionnaire**

A schematic of the mobile phone survey is found below. The MPS is comprised of several modular segments: (1) Language selection, (2) Introduction, (3) Demographic Screening, (4) Consent (5) NCD Modules and, (6) Incentive Delivery.

NCD modules are groups of questions on similar NCD behavioral risk factor topics covered in established NCD risk factor surveys such as WHO’s STEPS survey and the Global Adult Tobacco Survey (GATS). NCD modules include: tobacco use, alcohol consumption, dietary intake, physical activity, and current medications for blood pressure and diabetes.

To ensure that there will be an even distribution of answered NCD modules in those that do not complete the entire MPS, the order in which participants answer the NCD modules will be randomized, as illustrated in the figure below. For example, one participant may be presented NCD modules randomized in the following order: tobacco use, alcohol use, physical activity, dietary intake, and current medications for blood pressure and diabetes; while another participant may receive NCD modules in the order of physical activity, dietary intake, alcohol consumption, current medications for blood pressure and diabetes, and tobacco use. To preserve skip patterns programmed into the MPS, the order of questions within each module will not be randomized. The randomization of modules will occur in all *microtrials*. For the physical activity modules, participants will be randomized to receive one of two sets of physical activities questions: 1) International Physical Activity Questionnaires (IPAQ) or 2) Global Physical Activity Questionnaires (GPAQ).

**Figure 2. Components of the survey and randomization of NCD modules. The first several modular sequence will be fixed, and the NCD modules may be randomly ordered to prevent drop-off associated response bias.**


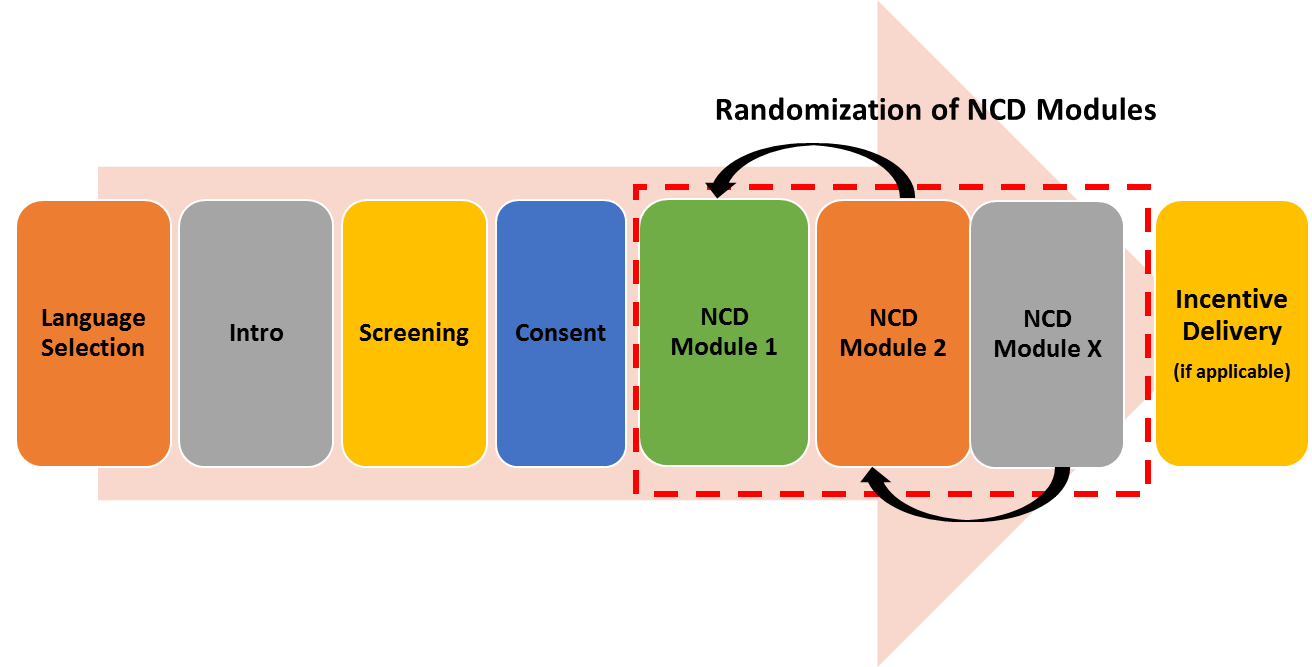


##

## **Survey Delivery**

The logistics and timing of MPS delivery will be similar for all *microtrials* with exceptions pointed out in each of the microtrials below. A brief description of the MPS delivery schema follows.

To help improve survey response and completion rates, an *SMS* *notification* will be sent to those who have been randomly selected to participate in the mobile phone survey. Previous studies have shown that notification SMS improve survey response and cooperation rates and decreases refusal rates.^11^ This SMS is meant to notify the potential respondent of the upcoming survey and will describe the purpose of survey, expected time commitment, and the survey’s sponsoring agency. The exact wording of the SMS and the timing of SMS delivery will be guided by focus group discussions (FGD) from a previous protocol (JHU IRB No: 0000709). Respondents will not be required to reply to the SMS in order to participate in the IVR survey; with the exception of *microtrial #2* (details can be found in the description of *microtrial#2)*.

The notification SMS will be sent before and on the same day on which the MPS will be sent. As discussed above, the exact timing of the notification SMS will be guided by the focus group discussions. Due to possible country specific mobile network problems, at a minimum, the SMS will be sent at least one hour before the MPS is sent; therefore ensuring the SMS reaches the participant before the IVR survey is sent. After the notification SMS is sent, the MPS will be sent to the same randomly generated mobile phone number. When a randomly dialed number is connected (i.e. a respondent picks up the phone), the respondent will be presented with a brief pre-recorded introduction to the study and provided an opportunity to select the survey’s language (except for *microtrial #5;* details provided below*)*. An example of the narrated introduction and language selection is, “Hello, its [Name] from the [Implementing Agency]. Earlier today, we sent you a text message about participating in a mobile phone survey.” After hearing the introduction, participants will be asked to select their preferred language for which the IVR survey will be narrated, “Please pick a language to continue hearing the survey instructions [and how you could qualify for free airtime (if countries are using airtime incentive] Press 1 to continue in [Language X] Press 2 to continue in [Language Y]” The results of the FGD (JHU IRB No: 0000709) will assist in the selection of the exact wording for the introduction and language selection.

After the language selection, the speaker will provide a more detailed description of the study which includes the purpose, duration, risks, benefits, and the sponsoring agency. If incentives are not prohibited in the country, participants will also be told about requirements to receive an incentive and the incentive amount. Potential respondents will be asked a demographic screening question. Respondents will be screened on age; those who indicate being less than 18 years old will be thanked for their interest and the survey will be terminated. If screening criteria are met, respondents will then be asked whether they agree to participate in the study (consent). Respondents who choose to continue with the survey - after hearing about its purpose, procedures and voluntary nature - will be asked to press a number on their keypad to indicate their willingness to continue (e.g. “Press 1 if you would like to participate in this survey. Press 3 if you do not want to participate in the survey”). Participants will be asked again to confirm their participation (e.g. “You have indicated that you do wish to complete the survey, press 1 to continue the survey or press 3 to end this survey”) to ensure that consent (or refusal) was not mistakenly provided. Respondents who do not agree to participate, will be thanked for their time and the survey will end. Following consent, respondents will receive and answer a series of NCD modules whose order of presentation is randomized as described above.

At any time after the MPS has been initiated (i.e. participants met screening criteria, consented, and began answering NCD topical questions) participants will be able to formally opt-out of survey participation by pressing a sequence of buttons on their mobile phone (The precise combination of buttons will be determined by in-country programming and implementation teams). NCD surveys will only be sent to participants once; that is participants who were ineligible due to screening criteria, refused consent, or formally opted out during the survey will not have an additional NCD survey sent.

The MPS will be delivered at random times ranging from 8:00am to 8:00pm local time. The exact time window to deliver the notification SMS and MPS will be determined by each country during the FGD (JHU IRB No: 0000709).

The potential survey outcomes are depicted in figure 3. In a large proportion of the randomly selected mobile phone numbers, the randomly dialed mobile phone number may not exist, participants may refuse to participate in the MPS, or the mobile phone number may be valid, but the IVR survey was not initiated. Reasons for this latter category include, (1) phone was turned off or out of battery, (2) mobile phone was out of network coverage area, and (3) personal reasons, which include not hearing the mobile phone ring or respondent hanging up before consenting to the study. In these instances where the MPS was not initiated by a respondent, no future call back attempts to enroll participant will be made. Moreover, for respondents who initiate the IVR survey, but do not complete it (labeled an ‘incomplete survey’ in the figure), no call back attempts to re-initiate the survey will be made. Note, that as described in the sample size section, a sub-sample of participants (n=30) will be called back by a human interviewer to ask why they did not complete the survey. Unlike traditional household surveys, multiple attempts to achieve participant response are not needed since the selection of mobile phone numbers and timing of delivery are both random, thereby reducing the potential for non-response bias, but also because of the very large pool of possible respondents in the sampling set of all mobile phone owners.

**Figure 3. IVR Survey Delivery Schedule and Possible Outcomes**

**
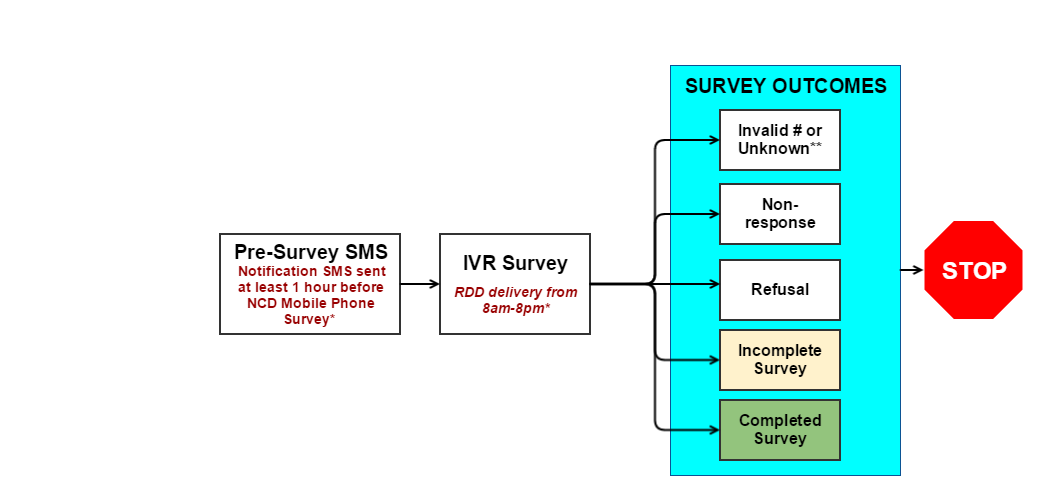
**

***** Timing of Notification SMS and window of survey delivery to be determined by FGD

**Microtrial Designs**

A description of each of the seven *microtrials* and the baseline IVR survey follows. Differences between the *microtrial* design and the IVR questionnaire and delivery (sections detailed above) will be pointed out. A broad description of the *microtrials* can be found in Table 2. Where participants have the opportunity to earn an airtime incentive, participants will be told of the requirements to earn this incentive during the survey’s introduction.

Objective 1: Evaluation of Incentives

*Baseline IVR* Survey

The purpose of the baseline IVR survey is to assess baseline completion rates to inform sample size calculations for the seven *microtrials*, to finalize the questionnaire used in IVR delivery, and to ensure that the IVR platform is functioning well (i.e. delivering SMS notifications, IVR surveys, and incentives correctly and as indicated in the protocol). For the baseline IVR survey, a 30 question containing survey will be sent to randomly dialed participants at random times of the day (See Appendix 1 for the questionnaire to be used). An airtime-based incentive of approximately $1.00 USD will be transferred to a respondent’s mobile phone if the survey is completed (i.e. all questions answered). The exact amount of airtime incentive will be guided by the finding of the formative FGD (JHU IRB No: 0000709)

IVR surveys will be pushed out by the system until 100 respondents have completed the survey. Overall contact, response, and completion rates will be calculated and applied to sample size calculations for the *microtrials*. The average time spent per question, in both completed and incomplete surveys, as well as average time spent to complete the survey will be tabulated. We anticipate that there will be a low completion percentage (i.e. all survey questions were answered) as participants may not want to spend a long time answering an IVR survey. We will assess the time point where between 40-60% of the participants terminate/drop-off from the IVR survey. For example, 50% of participants that initiate the IVR survey may stop the survey after answering questions for 10 minutes. For the subsequent *microtrials*, we will remove questions and NCD modules (i.e. shorten the questionnaire) until the average time spent is equal to the amount of time where 40-60% of participants drop-off from the survey. Technical glitches in survey delivery, incentive delivery, and sensitizing short message service (SMS) will also be assessed and resolved prior to commencement of the *microtrials.*

*Microtrial #1*- Evaluation of incentive amount

This *microtrial* seeks to examine whether there is a differential effect in completion rates based on incentive amount. This trial will be the first *microtrial* conducted. Participants will be randomized to receive one of three incentive amounts, in the form of airtime, which will be sent to participants upon completion of the IVR survey. The study arms are: 1) no incentive, 2) X USD incentive transferred after survey completion, where X is commiserate with the expected time commitment, daily wage, and its exact amount will be guided by FGDs and in consultation with country-partners, and 3) 2X incentive transferred after survey completion; where 2X is twice the amount of incentive in arm 2.

The response and completion rates will be examined by study arm to inform the incentive amount for *microtrial* #2. If there is an appreciable difference in response and completion rates between two incentive groups, the incentive amount that yields the highest response and completion rates will be used for *microtrial* #2. If there is no difference in response and completion percentages by incentive amount, the lower incentive (X) will be used for *microtrial* #2.

*Microtrial #2*- Evaluation of incentive timing

This microtrial seeks to examine whether the timing of incentive delivery (either given before the survey or afterwards) has an effect on contact, response, and/or completion rates. Participants will be randomized to one of three study arms: 1) no incentive, 2) a pre-paid airtime incentive; where 20-40%% of the total incentive (X) is sent to the respondent’s mobile phone after the sensitizing SMS and prior to initiation of the survey, and with the remaining 60-80% being sent after the survey’s completion (i.e. all questions answered), or 3) a promised incentive where 100% of the incentive amount is sent to the respondent’s mobile phone after the IVR survey is completed. With existing technology, the minimal amount of airtime that can be transferred is 1 USDThe total incentive amount (X) in this microtrial will be the incentive amount from *microtrial* #1 that produced the highest response and completion rates.

*Microtrial #3*- Evaluation of incentive structure

This microtrial seeks to examine whether the incentive structure (a fixed incentive amount or a lottery-based incentive) has an effect on response and completion rates. Respondents will be randomized to one of three study arms: 1) no incentive, 2) a fixed airtime incentive delivered after survey completion where the amount of the incentive is determined by *microtrial* 1, and 3) a lottery airtime incentive of an amount five times greater than the amount in the second study arm; where the odds of winning the lottery are 1:20.

Objective 2: Evaluation of the Surveys Introduction

*Microtrial #4*- Evaluation of content and voice of IVR survey’s introduction

This microtrial seeks to examine whether the content of the IVR survey’s introduction (motivational vs informational) and the survey’s narrative voice (male vs female) has an effect on contact, response, and/or completion rates. Participants will be randomized to one of four study arms with varying survey introduction content and voices: 1) male voice, informational content, 2) male voice, motivational content, 3) female voice, informational content, and 4) female voice, motivational content. Participants in each study arm will receive the same incentive.

*Microtrial #5*- Evaluation of a human operator IVR survey introduction

This microtrial seeks to examine whether the survey introduction’s modality (human operator versus those who receive an IVR survey introduction) has an effect on survey response and completion rates. Participants will be randomized to one of two arms: 1) a human will call the RDD-generated mobile phone number and read the survey’s introduction. The operator will answer any questions the participant may have, consent the participant and then patch the participant into the IVR survey, and 2) the participant will receive a IVR survey with a survey introduction in IVR format and similar to the previous *microtrials*.

Objective 3: Evaluation of the Sampling Frame

*Microtrial #6*- Evaluation of the sampling frame of mobile phone numbers

This microtrial seeks to examine whether the source of the mobile phone numbers has a differential effect on the IVR survey’s response and completion rates. The previous five *microtrials* relied on a RDD sampling frame. In this microtrial, we will obtain a de-identified list of mobile phone numbers from either a mobile network operator or from a previously conducted household survey where participants had consented to be contacted for additional studies. Participants will be sent an IVR survey with the top performing survey introduction (results from microtrials 4 and 5) and the top performing incentive (microtrials 1-3).

Objective 4: Evaluation of Survey Modality

*Microtrial #7*- Evaluation of CATI and IVR survey metrics

This microtrial seeks to examine whether the survey’s modality (IVR versus CATI) has a differential effect on the survey’s response and completion rates and to assess the ‘inter-modal reliability’. Participants will be randomized to one of two arms: 1) IVR then CATI or 2) CATI then IVR. Participants in the first study arm will receive an IVR survey first, followed by a CATI survey 7 days later. Participants in the second study arm will receive a CATI survey first followed by an IVR survey 7 days later. At initial enrollment, participants will be clearly explained that they are being enrolled in a microtrial where they will be contacted twice. The questionnaires used in both study arms will the same. This crossover design will allow for an assessment of response consistency, adjusted for the risk of ‘priming’ after exposure to the prior modality. This is the only *microtrial* that has participants answer two NCD surveys. The amount/timing/structure of incentive will be guided by the first three *microtrials*; where the incentive that yielded the highest response and completion rates will be provided to all participants in *microtrial* #7.

Follow-up of Non-response, Refusals, and Incomplete Interviews

For participants who were selected for the seven *microtrials,* a random sub-sample of participants who did not initiate the IVR survey, refused to participate, or partially completed an IVR survey, will be called back by a human operator using the same telephone number that was associated with the IVR. The study team will attempt to reach individuals up to three times. Unreached follow-ups will be documented. If successfully reached, the team will consent and interview participants to explore reasons for participant’s non-response, refusal, or incomplete surveys using a semi-structured guide (See Appendix 2). Demographic information from non-responders, refusals, and incomplete surveys will also be collected and examined in relation to the distribution of those who fully complete the survey.

**Table 2.** Study designs for Microtrials

|  | **Microtrial#1**  **Incentive Amount** | **Microtrial#2**  **Incentive Timing** | **Microtrial#3**  **Incentive Structure** | **Microtrial#4**  **IVR Intro features** | **Microtrial#5**  **IVR vs Human Intro** | **Microtrial#6**  **Sampling frame** | **Microtrial#7**  **Survey Modality** |
| --- | --- | --- | --- | --- | --- | --- | --- |
| **Population/Participants** | General mobile phone owning population | General mobile phone owning population | General mobile phone owning population | General mobile phone owning population | General mobile phone owning population | MNO mobile phone subscribers or Previous household survey sample | General mobile phone owning population |
| **Sampl-ing**  **Approach** | RDD | RDD | RDD | RDD | RDD | MNO- or household- provided | RDD |
| **Samp. Size** | -376 complete surveys per arm  -For 3 arms, n=1128 | -376 complete surveys per arm  -For 3 arms, n=1128 | -376 complete surveys per arm  -For 3 arms, n=1128 | -376 complete surveys per arm  -For 4 arms, n=1504 | -376 complete surveys per arm  -For 2 arms, n=752 | -376 complete surveys | -405 complete surveys per arm  -For 2 arms, n=752 |
| **Study Intervention** | No incentive | No incentive | No incentive | Male voice, informational intro | IVR Introduction | MNO- or household- provided | CATI |
|  | X USD airtime incentive post-IVR | 20% airtime pre-IVR; 80% airtime post-IVR | Fixed incentive of X post-IVR | Female voice, informational intro | Human Introduction |  | IVR |
|  | 2X USD airtime incentive post-IVR | 100% airtime post-IVR | Lottery incentive of 5X post-IVR | Male voice, motivational intro |  |  |  |
|  |  |  |  | Female voice, motivational intro |  |  |  |
| **Outcomes** | -CON, RR, COMP, R  -Attrition by question # and topic | -CON, RR, COMP, R  -Attrition by question # and topic | -CON, RR, COMP, R  -Attrition by question # and topic | -CON, RR, COMP, R  -Attrition by question # and topic | -CON, RR, COMP, R  -Attrition by question # and topic | -CON, RR, COMP, R  -Attrition by question # and topic | -CON, RR, COMP, R  -Attrition by question # and topic  -Reliability |
| **Notes** | -First microtrial to be conducted  -Incentive given after completion of IVR survey (Post-IVR) | -Incentive amount will range from X to 2X | -1:20 odds of winning lottery; incentive is 5 times amount in fixed incentive arm | -Provide same incentive in each arm  -The only difference in the questionnaire is the survey’s introduction. | -Human intro is a call center that asks participants if they wants to participate and if so, patches them into an IVR  -Provide same incentive in each arm | -Incentive and survey introduction used are top performers from microtrials #1-4 | - IVR arm will receive IVR survey first, followed by CATI survey 7 days later  -- CATI arm will receive a CATI first followed by an IVR survey 7 days later. |

Abbreviations: CON, contact rate; MNO, mobile network operator; RR, response rate, COMP, completion rate; R, refusal rate

***2. Describe the number and type of study visits and/or contacts between the study team and the participant, how long they will last, and where/how they will take place.***

There will be no face-to-face (i.e. in-person) contact between participants and the study staff. All interactions with research participants will be through the participant’s personal mobile phone. Participants will be randomly selected from a list of randomly generated mobile phone numbers (microtrials 1-5) or from a pre-defined and de-identified list of mobile numbers provided by a MNO or household survey. The selected mobile phone number will be sent one text message that alerts the potential participant about the upcoming survey. For all *microtrials* except for *microtrial #7,* participants will be sent one MPS. If a participant decides to answer the MPS, the time to complete the survey should be no more than 30 minutes. For *microtrial #7*, participants will be sent a CATI and IVR survey separated by seven days. In a sub-sample of participants who either refused to participate in the IVR survey, did not initiate the survey, or who did not complete the survey, participants will be called back by a human operator to understand reasons for their not fully participating in the survey. This interview is expected to take no more than 30 minutes. Therefore, participants may have up to three contacts with the mobile phone survey. For each contact, a verbal consent will be taken.

***3. Describe the expected duration of the study from the perspective of the individual participant and duration overall.***

A MPS is expected to take no more than 30 minutes. Within one week of being sent an MPS, a sub-sample of participants may be called back by a human operator to understand reasons why they did not fully participate in the survey. Therefore for microtrials #1-6, the potential length of duration would be one week with up to two study contacts. For *microtrial* #7, participants will be sent two surveys spaced seven days apart. Following the second survey, a sub-sample of participants may be called back to understand reasons why they did not fully participate in the survey. Therefore, for *microtrial #7* the potential length of duration would be two weeks with up to three study contacts.

***4. Provide a brief data analysis* plan *and a description of variables to be derived.***

The contact rates, response rates, and completion rates will be tabulated for each of the microtrials by study arm; where contact, response, and completion rates are defined as:

The contact, response, and completion proportions^[[1]](#footnote-1)^ will be tabulated for each of the *microtrials* and by study arm where contact, response, and completion are defined as:

$$Contact Rate= \frac{CI+PI+R}{CI+PI+R+(NC+U)}$$

$$Response Rate= \frac{CI+PI}{CI+PI+R+NC}$$

$$Completion rate= \frac{\mathrm{CI}}{CI+PI+R}$$

**CI** (Complete Interview): a respondent that answered all applicable questions

**PI** (Partial interview): a respondent who consented to the survey but did not answer all questions

**R** (Refusal): a respondent who: 1) did not consent to the survey, or 2) formally withdrew during the survey

**NC** (Non-contact): a working phone number where the respondent picked up but did not engage with the survey. This is assessed by looking at the system log for time spent on the survey

**U** (Unknown): a phone number that cannot be determined whether it is a working phone number (the respondent did not pick up the phone) or not (the phone number is not eligible)

Respondents who report being less than 18 years old are considered ineligible. Ineligible respondents are excluded from the equations for contact, response, and completion rates.

Contact, response, and completion proportions will be compared by study arm for each microtrial and by incentive group. *A priori, a*n alpha of 0.05 will be used for hypothesis testing.

Partially completed surveys will be tabulated and stratified by both the question number, and the question content due to the randomization of NCD topic modules. Additionally, the average time spent answering, the number of “Don’t know” or “Refuse to answer” responses, and the number of times the question was repeated will be calculated for both the chronological question number and question content and stratified by study arm. For microtrial #7, test for agreement in survey responses between the survey modalities will be assessed using Cohen’s Kappa.

In microtrial #7, participants will be asked to answer two surveys (IVR and call center [CATI]) composed of the same questions so that question validity can be assessed. For example, analyses will be conducted to observe whether some people may be more likely to report unhealthy habits when taking the automated IVR survey as compared to a call center because the human element is removed in an IVR survey.

Risk factor data will be collected and stored from other microtrials. This data will be compared to publically available results and national estimates to help determine if the protocol’s mobile-collected risk factor’s prevalence are similar to national estimates of household collected data. This will help answer whether the protocol’s mobile phone sample is as healthy, or unhealthy, as the traditionally administered surveys.

*5.*  ***Describe whether you are collecting or storing personal identifiers, and if yes, why you need them, and when and how you plan to dispose of them. Signatures on consent forms are considered to be identifiers.***

We will not collect personal identifiers via the consent process. Mobile phone numbers that were randomly generated are the personal identifiers. Because of the nature of the trial, the mobile phone numbers are needed to generate data. While phone numbers will initially be logged in the VOTOmobile platform alongside the data collected, immediately after each microtrial has been completed, the PI will download the database from the VOTOmobile platform and permanently delete the column containing mobile phone numbers. Phone numbers need not be retained for purposes of the study. No other potentially individually identifying data will be collected.

**VI. Data Custody, Security, and Confidentiality Protections**

*The sections below describe types of data sources and how they will be protected. For the type(s) of data you will have, put an “X” in the appropriate box to the left of the section that best describes how you will minimize the risk of a breach of confidentiality for your study. Note, as appropriate, how you will record/store data. These descriptions represent MINIMAL measures; you may add more stringent protections and other relevant information in B*.

**Confidentiality: The *LOSS OR THEFT* of 1) original/duplicate version of physical data collection instruments (forms, tapes, etc) or 2) physical devices containing electronic data (i.e. laptop/mobile device, external flash drive(s), is a threat to subject confidentiality. Risk of such a loss/theft is increased during movement/transport of data (in any format), such as in a vehicle or other move. Be sure to train anyone (co-investigators, staff, students, etc.) who might be engaged in the oversight of data handling/storage about this problem. Some typical risk-mitigation strategies would include:**

- **minimizing the physical movement of data and/or devices containing data**
- **encrypting electronic data (especially when stored on any mobile device, including flash memory tools, phones, tablets, etc, or when transferring across networks)**
- **making use of reliable courier services (FedEx, DHL, etc) when physical transport of bulk data forms is necessary**
- **minimizing the transfer of identifiable data in physical or electronic form (i.e. removing/separating/destroying identifiable data, when physical transfer of data is necessary)**

A. Data Storage

| 1. Hard Copies of Data Collection Forms. | |
| --- | --- |
| X | This activity will not involve receiving and/or accessing hard copies of data |
|  | Data collection forms RECORD NO PERSONAL IDENTIFIERS connecting study participants, and there are no codes providing a link. Data are anonymous. |
|  | Data collection forms INCLUDE IDENTIFIERS. The forms are locked in a secure cabinet or room with limited access by authorized individuals. Forms will be kept in study team’s possession during transport and will not be left unattended in a vehicle. When possible, de-identified copies will be used for coding and analysis. |
|  | Data collection forms ARE CODED with study participants’ random study ID numbers. Codes/links between study IDs and identifiers are stored securely in a separate place (locked storage cabinet or secure electronic database.) |
|  | Other: |
| 2. Electronic Data | |
|  | The data do not contain personally identifiable information |
|  | These data are stored on a secure server protected by limited access and strong password systems. Data are coded when possible. Portable electronic devices will not contain identifiable information unless encrypted. |
| X | Other: Phone numbers will be logged in a security encrypted and password protected VOTOmobile platform alongside other non-identifiable data. Immediately after each microtrial has been completed, the PI will download the database from the VOTOmobile platform and permanently delete the column containing mobile phone numbers. |
| 3. Other Identifiable Data Storage, Retention, and Destruction (Audiotapes, videotapes, photographs, etc.) will be retained and stored securely (locked in cabinet or room) until: **NA** | |
|  | Transcription is complete, then will be destroyed. |
|  | Analysis is complete, then will be destroyed. |
|  | Study is complete and file is closed. |
|  | Indefinitely. Provide justification for indefinite retention: |
| 4. Existing Biospecimens to be used in this study: **N/A** | |
|  | HAVE NO PERSONAL IDENTIFIERS. |
|  | INCLUDE IDENTIFIERS AND ARE CODED; the PI will not have access to the link or code connecting the identifiers to the specimens. |
|  | INCLUDE IDENTIFIERS, and the PI has access to those identifiers or to the link/code connecting specimens to individuals. The identifiers and/or code will be stored securely until the study is complete. |

B. **Certificate of Confidentiality**

***Will the study data stored in the United States be protected by a Certificate of Confidentiality? If yes, explain who will apply for and maintain the Certificate.*** (<http://grants.nih.gov/grants/policy/coc/appl_extramural.htm>)

No

C. **Data Security and Sharing**

***PIs have the responsibility for responsible stewardship of data and protecting data confidentiality. This responsibility includes protecting physical custody of the data, storage and sharing with appropriate data use agreements that contain the appropriate security provisions. Describe any additional plans beyond those identified in the table that you have for storing and sharing the study data and/or materials, and how responsibility for the data will be managed. Include the following details:***

*1.* ***Where will the study data be stored?***

*2.* ***Who controls access to the data?***

*3.* ***Will data be shared only if de-identified?***

*4.* ***What additional (if any) security controls will be in place*?**

Electronic data (survey results) will be downloaded from VOTOmobile’s cloud platform onto a password protected computer of the study staff, located behind a locked door at JHSPH. As above, these data will permanently de-identified by stripped them of phone numbers. Only members of the JHSPH study team will have access to these data. Data shared with partner countries will also be de-identified and maintained in locked filing cabinets and on password protected computers. Country investigators will follow data security protocols of their home institutions.

**VII. Risks of the Study**

***A. Describe the risks, discomforts, and inconveniences associated with the study and its procedures, including physical, psychological, emotional, social, legal, or economic risks, and the risk of a breach of confidentiality. These risks should be described in the consent documents.***

There are no foreseeable physical risks to study participants. During the IVR survey, some participants may feel uncomfortable or bored while answering questions. Minor psychological discomfort may be associated with responding to questions of a personal nature on habits regarding socially undesirable behaviors such as use of tobacco products, alcohol, or unhealthy diet and physical inactivity.

A limited number of questions will be asked of individuals to minimize inconvenience and boredom. The survey is expected to last no longer than 30 minutes. To minimize risks of psychological discomfort, participants will be informed that they do not need to answer any question that they feel uncomfortable with and that their participation in the survey is voluntary. As the IVR survey will collect responses from individuals non-verbally (i.e., by pressing numbers that correspond with answer choices), the likelihood of responses being known to others who may be in their vicinity is also decreased.

*B.* ***Describe the anticipated frequency and severity of the harms associated with the risks identified above; for example, if you are performing “x” test/assessment, or dispensing “y” drug, how often do you expect an “anticipated” adverse reaction to occur in a study participant, and how severe do you expect that reaction to be?***

We do not know precisely the frequency and severity of potential discomfort but anticipate that it will be quite infrequent and minimal given the nature of the survey questions.

*C.* ***Describe steps to be taken to minimize risks. Include a description of your efforts to arrange for care or referral for participants who may need it.***

To minimize risks associated with answering questionnaires, participants will be informed that they do not need to answer any question that they feel uncomfortable with and that their participation in the IVR survey is voluntary. Participants will be informed and allowed to end their participation at any time. To minimize risk of confidentiality breach, we have employed several safeguards. First, the IVR survey does not collect any personal identifying information aside from the mobile phone number (date of birth and name are not collected). Following completion of the IVR surveys, the PI will download data from the VOTOmobile platform and permanently delete the column containing mobile phone numbers. After this step, there will be no record of mobile phone numbers associated with the IVR survey.

No names will be used in any reports or documents stemming from this research protocol. Study staff who are involved with microtrial 5 and microtrial 7 (human operators) and who call back participants to understand reasons for their non-participation will be trained in human research ethics.

*D.* ***Describe the research burden for participants, including time, inconvenience, out-of pocket costs, etc.***

Overall, participants will spend up to 30 minutes for each mobile phone survey in the seven microtrials. For microtrial seven, participants receive two mobile phone surveys. This amount of time may be inconvenient for participants. In a sub sample of microtrial participants, we will call individuals to understand reasons for their non-participation; at most, this would be 30 minutes. The costs of airtime associated with answering the MPS will be borne by the study team and transferred directly to respondents within 24 hours of their completing a survey. VOTOmobile has experience with issuing airtime credit transfers across multiple mobile network providers. Therefore, participants will not incur any out-of-pocket expenses as part of their participation in the study.

*E.* ***Describe how participant privacy will be protected during data collection if sensitive questions are included in interviews.***

The survey questions are administered over a telephone call. Therefore, it will be difficult for people not associated with the study to hear and understand the survey questions and participant’s responses.

**VIII. Direct Personal and Social Benefits**

*A.* ***Describe any potential direct benefits the study offers to participants (“payment” for participation is not a direct personal benefit).***

Participants will not have any direct benefit from their participation.

***B. Describe potential societal benefits likely to derive from the research, including value of knowledge learned.***

Input received about MPS and NCD data collection will be used to improve future implementation of NCD surveillance efforts and if surveillance efforts are improved, the initiative may lead to better health monitoring, health policies and health outcomes for people living in the countries involved in this study.

**IX. Payment:**

***A. Describe the form, amount, and schedule of payment to participants. Reimbursement for travel or other expenses is not “payment,” and if the study will reimburse, explain.***

***B. Include the possible total remuneration and any consequences for not completing all phases of the research.***

Participants will be provided an airtime incentive as described in the study design. The incentive timing and amount varies by microtrial and study arm. Aside from the airtime incentives described in the microtrials, no other airtime incentive or payment will be provided. No payment will be sent to participants selected for completing the small follow-up study to understand reasons for non-participation.

**X. Study Management**

A. **Oversight Plan:**

1. ***Describe how the study will be managed.***

The Principal Investigator, Dr Adnan Hyder, will be responsible for overseeing the JHSPH team’s involvement in the study and coordinating with all partners. JHSPH co-investigators will work with Dr Hyder and country teams to support implementation of the protocol. Dr Dustin Gibson will provide technical support and coordinate JHSPH activities. Dr George Pariyo will provide technical support and coordinate country engagement activities. Dr Alain Labrique will provide technical support. Mr Joseph Ali will provide technical support and analyses related to ethics and regulatory components. Dr Saifuddin Ahmed will provide statistical assistance with sampling and analysis of de-identified data. **No members of the JHSPH study team will have contact with human participants.**

***2. What are the qualifications of study personnel managing the project?***

Adnan Hyder is a Professor and Director of the Health Systems program within the IH Department, JHSPH. He is the principal investigator of a Bloomberg Philanthropies grant that seeks to assess the validity of national level IVR estimates of NCD risk factors.

Dustin Gibson received his PhD from JHSPH and is an Assistant Scientist in the Health Systems Program. He has extensive experience developing and evaluating mHealth technologies. He was the study coordinator for a village randomized controlled trial that used mobile phone technologies to improve immunization in western Kenya.

George Pariyo received his PhD from JHSPH and is a Senior Scientist in the Health Systems Program. He has extensive experience conducting implementation research on health systems.

Alain Labrique is an Associate Professor in the Global Disease Epidemiology and Control Program within the Department of International Health. He is also the founding director of the Johns Hopkins University Global mHealth Initiative; a university-wide consortium that strives to develop and evaluate mobile health and ICT technologies.

Joseph Ali is a lawyer and Research Scholar in the Johns Hopkins Berman Institute of Bioethics and Associate Faculty in the JHSPH Department of International Health. He has expertise in international research ethics, IRB coordination, and the conduct of collaborative research.

Saifuddin Ahmed is an Associate Professor in the Department of Population, Family & Reproductive Health with a joint appointment in Biostatistics. He received his PhD from JHSPH and is trained in demography and epidemiology. He has expertise in NCD epidemiology, quantitative research methods and complex population surveys.

***3. How will personnel involved with the data collection and analysis be trained in human subjects research protections? (Use the JHSPH Ethics Field Training Guide on our website.)***

All JHSPH study personnel will have undergone human research ethics training and have completed the CITI course. The JHSPH Ethics Field Training Guide and CITI module will be recommended to country partners as a useful reference, and all country teams will complete human subjects training as required by their institutions and countries. Country PIs will ultimately be responsible for ensuring that their study teams are sufficiently trained in human subjects research.

***4. If the PI will not personally be on-site throughout the data collection process, provide details about PI site visits, the supervision over consent and data collection, and the communication plan between the PI and study team.***

Local country PIs will be on site throughout data collection processes. The JHSPH PI and co-investigators will participate in regularly scheduled conference calls (at least once a month) with in-country PI’s and email correspondence as needed. JHSPH investigators, on request by in-country partners, will visit countries to provide technical support. We will maintain close daily/weekly communication via email and phone with VOTOmobile before and during data collection. The data collection activities described herein build directly upon existing country collaborations and formative work described under a separate JHSPH approved protocol (IRB No: 0000709). Consent and data collection will be automated for the IVR surveys and scripted for the CATI.

**B. Recordkeeping:**

***Describe how you plan to ensure that the study team follows the protocol and properly records and stores study data collection forms, IRB regulatory correspondence, and other study documentation. For assistance, contact*** [***housecall@jhsph.edu***](mailto:housecall@jhsph.edu)***.***

Procedures for maintenance of all IRB regulatory correspondences, study documentation, and de-identified data kept at JHSPH will be reviewed by the JHSPH study team at the outset of the study and at least once per year thereafter. The JHSPH PI will be responsible for ensuring compliance with protocol for components that involve JHSPH. The JHSPH study team will also review recordkeeping best practices with country teams and VOTOmobile prior to study initiation as a reminder that it is essential to maintain certain study records and correspondences, and to do so securely and in accordance with protocols.

C. **Safety Monitoring**

N/A

**D. Reporting unanticipated problems/adverse events (AE’s) to the IRB**

***Describe your plan for reporting to the IRB and (if applicable) to the sponsor. Include your plan for government-mandated reporting of abuse or illegal activity. NOTE: The IRB does not require submission for all AEs, only those that are unanticipated, pose risk of harm to participants or others, and are related to the study.***

In the unlikely scenario where an AE occurs, the investigator to whom an AE is reported will immediately consult with the PI and other members of the study team. If warranted, the PI will contact the IRBs of record to provide a detailed account of the AE. Any AE that is related to the study, is unanticipated, and poses risk of harm to participants or others will be reported to the JHSPH IRB.

**E. Other IRBs/Ethics Review Boards:**

***If other IRBs will review the research, provide the name and contact information for each IRB/ethics review board and its Federal Wide Assurance, if it has one (available on OHRP’s website at*** [***http://www.hhs.gov/ohrp/assurances***](http://www.hhs.gov/ohrp/assurances)***).***

During this optimization phase, each country will independently develop and maintain their own local study protocols and will follow local procedures for obtaining IRB review and approval of their protocols. As country engagement is ongoing, we are not able to list all IRBs that will be involved in reviewing local protocols.

**F. Collaborations with non-JHSPH Institutions:**

***For studies that involve collaboration with non-JHSPH institutions, complete the chart below by describing the collaboration and the roles and responsibilities of each partner, including the JHSPH investigator. This information helps us determine what IRB oversight is required for each party. Complete the chart for all multi-collaborator studies.***

**Insert Name of Institutions in Partner column(s); add additional columns if necessary.**

|  | JHSPH | Countries (n=4) | VOTO-mobile |
| --- | --- | --- | --- |
| Primary Grant Recipient | X |  |  |
| Collaborator | X | X | X |

**For the following, indicate “P” for “Primary”, “S” for “Secondary” as appropriate to role and level of responsibility.) Add additional items if useful.**

| 1 | Human subjects research ethics training for data collectors | S | P | P |
| --- | --- | --- | --- | --- |
| 2 | Day to day management and supervision of data collection | S | P | P |
| 3 | Reporting unanticipated problems to the JHSPH IRB/Sponsor | S (collaborator to notify JHSPH team) | P | P |
| 4 | Hiring/supervising people obtaining informed consent and/or collecting data |  | P | P |
| 5 | Execution of plan for data security/protection of participant data confidentiality, as described in Sect. 5. | S (related to handling de-identified data) | P | P |
| 6 | Biospecimen processing, storage, management, access, and/or making decisions about future use | N/A | N/A | N/A |

**COMPLETE THE FOLLOWING SECTIONS WHEN RELEVANT TO YOUR STUDY:**

**XI. Secondary Data Analysis of Existing Data: N/A**

**XII. Oversight plan for student-initiated studies: N/A**

**XIII. Creation of a biospecimen repository: N/A**

**XIV. Data Coordinating Center: N/A**

**XV. Drug Products, Vitamins, Food and Dietary Supplements: N/A**

**XVI. Investigational Medical Devices: N/A**

REFERENCES

1. GBD 2013 DALYs and HALE Collaborators, Murray CJ, Barber RM, et al. Global, regional, and national disability-adjusted life years (DALYs) for 306 diseases and injuries and healthy life expectancy (HALE) for 188 countries, 1990-2013: quantifying the epidemiological transition*. Lancet*. 2015. doi: S0140-6736(15)61340-X [pii].

2. GBD 2013 Mortality and Causes of Death Collaborators. Global, regional, and national age-sex specific all-cause and cause-specific mortality for 240 causes of death, 1990-2013: a systematic analysis for the Global Burden of Disease Study 2013*. Lancet*. 2015;385(9963):117-171. doi: 10.1016/S0140-6736(14)61682-2 [doi].

3. Global Burden of Disease Study 2013 Collaborators. Global, regional, and national incidence, prevalence, and years lived with disability for 301 acute and chronic diseases and injuries in 188 countries, 1990-2013: a systematic analysis for the Global Burden of Disease Study 2013*. Lancet*. 2015;386(9995):743-800. doi: 10.1016/S0140-6736(15)60692-4 [doi].

4. Noncommunicable diseases Fact Sheet. <http://www.who.int/mediacentre/factsheets/fs355/en/>.

5. World Health Organization. *2008–2013 Action Plan for the Global Strategy for the Prevention and Control of Non-Communicable Diseases*. 2010.

6. STEPS Manual. <http://www.who.int/chp/steps/manual/en/>.

7. Barr ML, van Ritten JJ, Steel DG, Thackway SV. Inclusion of mobile phone numbers into an ongoing population health survey in New South Wales, Australia: design, methods, call outcomes, costs and sample representativeness*. BMC Med Res Methodol*. 2012;12:177-2288-12-177. doi: 10.1186/1471-2288-12-177 [doi].

8. Liu B, Brotherton JM, Shellard D, Donovan B, Saville M, Kaldor JM. Mobile phones are a viable option for surveying young Australian women: a comparison of two telephone survey methods*. BMC Med Res Methodol*. 2011;11:159-2288-11-159. doi: 10.1186/1471-2288-11-159 [doi].

9. Potthoff RF. Telephone sampling in epidemiologic research: to reap the benefits, avoid the pitfalls*. Am J Epidemiol*. 1994;139(10):967-978.

10. International Telecommunications Union. The World in 2015: ICT Facts and Figures. 2015.

11. Steeh C, Buskirk TD, Callegaro M. Using text messages in U.S. mobile phone surveys*. Field Methods*. 2007;19(1):59-75.

1. Adapted from: The American Association for Public Opinion Research. 2015. *Standard Definitions: Final Dispositions of Case Codes and Outcome Rates for Surveys. 8^th^ edition.* AAPOR. [↑](#footnote-ref-1)
